# Nutraceuticals in Psychiatric Disorders: A Systematic Review

**DOI:** 10.3390/ijms25094824

**Published:** 2024-04-28

**Authors:** Paola Bozzatello, Roberta Novelli, Cristiana Montemagni, Paola Rocca, Silvio Bellino

**Affiliations:** Department of Neuroscience, University of Turin, Via Cherasco 15, 10126 Turin, Italy; roberta.novelli@unito.it (R.N.); cristiana.montemagni@unito.it (C.M.); paola.rocca@unito.it (P.R.); silvio.bellino@unito.it (S.B.)

**Keywords:** nutraceuticals, omega-3 fatty acids, probiotics, psychiatric disorders

## Abstract

Correct nutrition and diet are directly correlated with mental health, functions of the immune system, and gut microbiota composition. Diets with a high content of some nutrients, such as fibers, phytochemicals, and short-chain fatty acids (omega-3 fatty acids), seem to have an anti-inflammatory and protective action on the nervous system. Among nutraceuticals, supplementation of probiotics and omega-3 fatty acids plays a role in improving symptoms of several mental disorders. In this review, we collect data on the efficacy of nutraceuticals in patients with schizophrenia, autism spectrum disorders, major depression, bipolar disorder, and personality disorders. This narrative review aims to provide an overview of recent evidence obtained on this topic, pointing out the direction for future research.

## 1. Introduction

Over the past decades, growing evidence has been obtained that proper nutrition with high intakes of nutrients such as fibers, phytochemicals, and short-chain fatty acids (omega-3 fatty acids) is correlated with mental health and prevention of neurodevelopmental disorders [1,2,3,4,5].

The effects of omega-3 fatty acids on inflammatory processes, the cardiovascular system, and the nervous system are recognized by many studies [1,3,4,6]. Polyunsaturated omega-3 fatty acids are important components of phospholipids and cholesterol esters of the neuronal cell membrane, especially of dendritic and synaptic membranes. In the brain, these agents modulate brain cell signaling, involving dopaminergic and serotonergic pathways [2,7,8]. They produce modifications of the phospholipid fatty acid composition of the synaptic membrane and modulate the cascade of second messengers [9,10,11]. In particular, eicosapentaenoic acid (EPA) and docosahexaenoic acid (DHA) are significant regulating factors of neurogenesis, cell survival, and neurotransmission [3,12].

Also, the human gut microbiota, through the secretion of short-chain fatty acids, which can modulate tryptophan availability and vagal activation, can alter inflammatory signaling in the brain [13]. Therefore, changes in the microbiome may affect cognitive ability and behavior [13,14]. A strong relationship between microbiome status and neurocognitive states has been reported [15]. Furthermore, changes in the activity of different brain regions in response to changes in the microbiome suggest that the human microbiome and associated products are important determinants of neuronal coordination [16].

Several studies have shown that neuropsychiatric and neurodegenerative disorders such as major depressive disorder, bipolar disorder, Parkinson’s disease, Alzheimer’s disease, functional disorders, and autoimmune disorders such as multiple sclerosis are linked to neuroinflammation and that their onset and regulation depend on certain physical factors, including the microbiome [13,17,18]. 

Based on these findings, omega-3 was also studied as a potential treatment for several psychiatric disorders such as schizophrenia, major depression, bipolar disorder, autism spectrum disorders, and personality disorders [12,19,20,21,22,23,24]. Concerning the effects of nutraceuticals on psychiatric symptoms and/or disorders, data are still limited. Existing evidence pointed out that supplementation with omega-3 fatty acids provides benefits in the main psychiatric symptom dimensions, particularly in affective symptoms, impulsivity, and harmful behaviors [25,26,27].

This narrative review aims to provide an updated account of the available evidence of the impact of omega-3 fatty acids and other nutraceuticals on psychopathology in patients with schizophrenia, autism spectrum disorder, major depression, bipolar disorder, and personality disorders. The objective is to establish whether data collected in trials of omega-3 fatty acids and nutraceuticals in the treatment of psychiatric disorders support their indications in treating patients with specific diagnoses.

## 2. Methods

**Figure 1:**
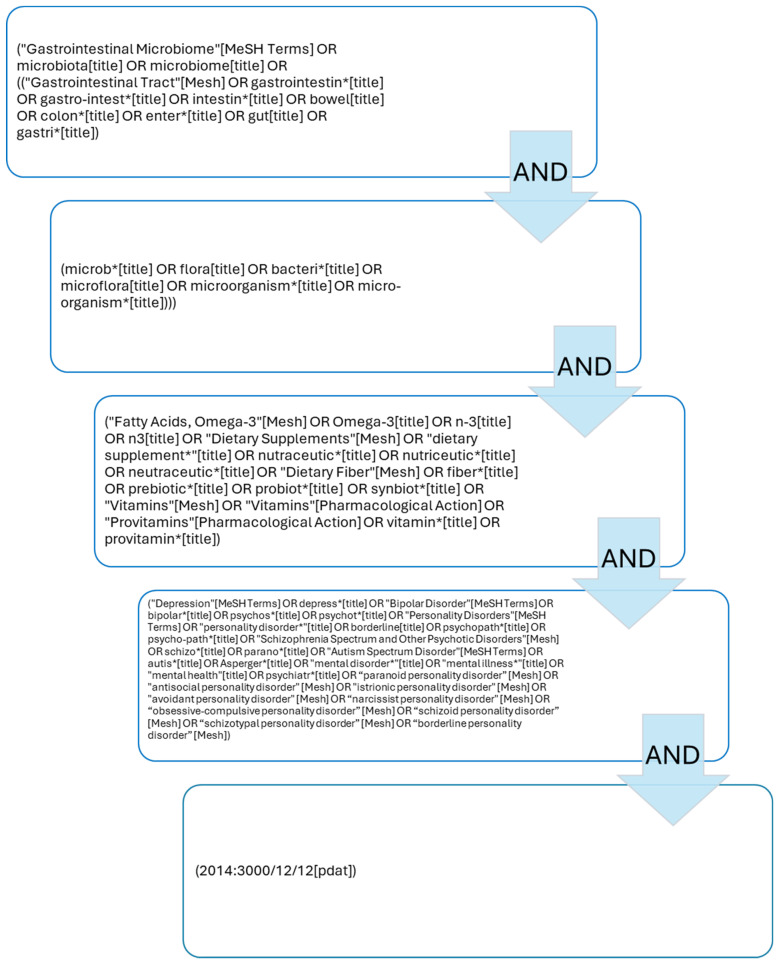
Search string on PubMed.

In January 2024, an electronic search was performed on PubMed on the role of nutraceuticals, probiotics, and omega-3 fatty acid supplementation in the treatment of psychiatric disorders such as personality disorders, schizophrenia, bipolar disorder, major depression, and autism spectrum disorders using the following search string in PubMed (“Gastrointestinal Microbiome”[MeSH Terms] OR microbiota[title] OR microbiome[title] OR ((“Gastrointestinal Tract”[MeSH] OR gastrointestin*[title] OR gastro-intest*[title] OR intestin*[title] OR bowel[title] OR colon*[title] OR enter*[title] OR gut[title] OR gastri*[title]) AND (microb*[title] OR flora[title] OR bacteri*[title] OR microflora[title] OR microorganism*[title] OR micro-organism*[title]))) AND (“Fatty Acids, Omega-3”[MeSH] OR Omega-3[title] OR n-3[title] OR n3[title] OR “Dietary Supplements”[MeSH] OR “dietary supplement*”[title] OR nutraceutic*[title] OR nutriceutic*[title] OR neutraceutic*[title] OR “Dietary Fiber”[MeSH] OR fiber*[title] OR prebiotic*[title] OR probiot*[title] OR synbiot*[title] OR “Vitamins”[MeSH] OR “Vitamins”[Pharmacological Action] OR “Provitamins”[Pharmacological Action] OR vitamin*[title] OR provitamin*[title]) AND (“Depression”[MeSH Terms] OR depress*[title] OR “Bipolar Disorder”[MeSH Terms] OR bipolar*[title] OR psychos*[title] OR psychot*[title] OR “Personality Disorders”[MeSH Terms] OR “personality disorder*”[title] OR borderline[title] OR psychopath*[title] OR psycho-path*[title] OR “Schizophrenia Spectrum and Other Psychotic Disorders”[MeSH] OR schizo*[title] OR parano*[title] OR “Autism Spectrum Disorder”[MeSH Terms] OR autis*[title] OR Asperger*[title] OR “mental disorder*”[title] OR “mental illness*”[title] OR “mental health”[title] OR psychiatr*[title] OR “paranoid personality disorder”[MeSH] OR “antisocial personality disorder”[MeSH] OR “istrionic personality disorder”[MeSH] OR “avoidant personality disorder”[MeSH] OR “narcissist personality disorder”[MeSH] OR “obsessive-compulsive personality disorder”[MeSH] OR “schizoid personality disorder”[MeSH] OR “schizotypal personality disorder”[MeSH] OR “borderline personality disorder”[MeSH]) AND (2014:3000/12/12[pdat]). The search string is displayed in the Figure 1.

We included the following types of publications: randomized controlled trials (RCTs), follow-up studies, open-label trials, proof-of-concept studies, posthoc subgroup analyses, naturalistic follow-up studies, pilot studies series, case studies, narrative reviews, systematic reviews, meta-analysis from January 2014 to February 2024. We have excluded observational studies, longitudinal studies, prospective studies, and letters to the author. Overlapping studies were also excluded.

Eligibility status for articles was determined in the following way: (1) all studies were screened based on the title and abstract and (2) papers that passed the initial screening were reviewed based on a careful examination of the full manuscript content. The review considered only articles written in English.

To make the review as comprehensive as possible, all articles with a cohort size between 1 and 377 patients were selected; patients in whom the psychiatric disorder had already been diagnosed and subjects at risk were examined; the observation period was between 3 and 260 weeks. No age limits were set. Studies were examined in which a single agent was evaluated against a placebo and studies in which two or more compounds were compared with each other, with or without the combination of psychiatric medications. No ethnicity was excluded. Details of the studies are reported in the tables [Table 1, Table 2, Table 3, Table 4 and Table 5].

## 3. Results

**Figure 2:**
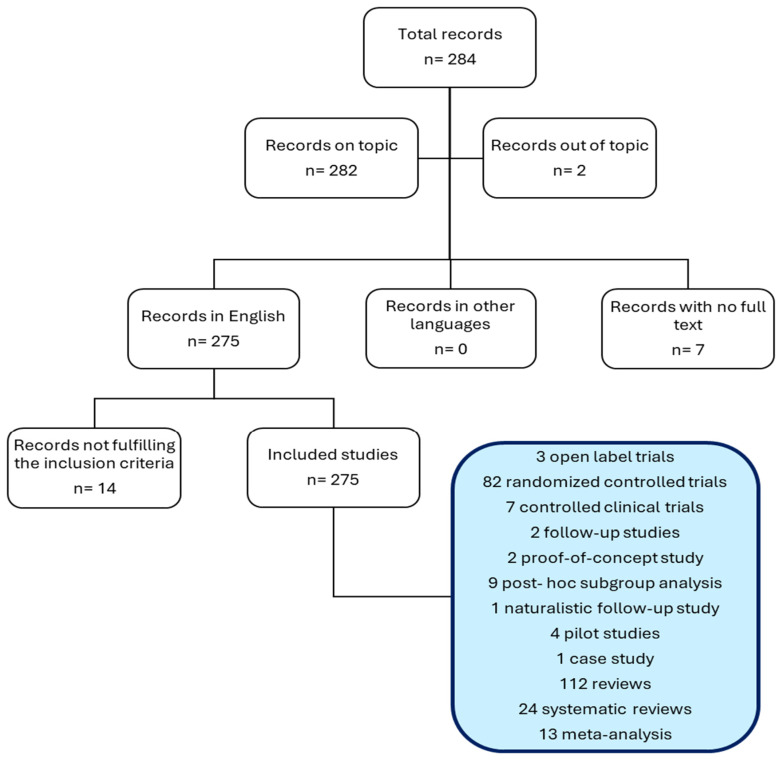
Literature search flowchart.

Records from PubMed and study screening are displayed in the following flowchart (Figure 2).

## 4. Discussion

### 4.1. Schizophrenia

Schizophrenia is a psychotic disorder with a clinical picture defined by five symptom domains: delusions, hallucinations, disorganized thinking, disorganized behaviors, and negative symptoms. The syndrome must be present continuously for more than six months and has usually a chronic course [135,136,137]. To evaluate the effects of omega-3 fatty acids and nutraceuticals in the different phases of schizophrenia, investigations were conducted in subjects at high risk of developing psychosis, patients with their first psychotic episodes, and patients with chronic phases.

Studies indicated that the etiology and severity of schizophrenia may be influenced by genetic abnormalities in the metabolism of fatty acids, prostaglandins, and phospholipids [138,139]. They were observed in the early phases of the disease also before the onset of psychotic symptoms [140]. In both chronic and non-medicated first-episode patients with psychosis, unsaturated fatty acid levels were found to be significantly reduced in the erythrocyte membranes and the post-mortem brain tissue in comparison to healthy controls [141,142,143,144,145]. A decrease in the proportion of omega-3 fatty acids in the cellular membranes was also associated with worse functioning before the onset of psychosis [145] and could be linked with greater severity of symptoms and poor therapeutic response [146,147]. A significant relationship was observed between lower erythrocyte essential fatty acid concentration and greater severity of negative symptoms [140,148], cognitive impairment, and tardive dyskinesia [149]. 

In the past, it was observed that between 22% and 40% of adolescents and young adults who are classified as high-risk or ultra-high-risk (UHR) of psychosis underwent a transition to psychosis within three years after the first examination [150,151,152]. These data indicate the need for diagnostic and therapeutic interventions to prevent the onset of psychosis [153]. Some authors [145] analyzed the correlation between metabolic abnormalities and the risk of psychosis (evaluated using the Scale of Prodromal Symptoms (SOPS) from the Structured Interview for Prodromal Symptoms (SIPS)) and found that over 90% of high-risk subjects in the cohort showed elevated oxidative stress. They found a relatively low (<4%) red blood cell omega-3 fatty acids index and a significant association between cardiometabolic abnormalities, lower levels of omega-3 fatty acid intakes, and increased severity of symptoms and impairment of functioning, suggesting that dietary factors and systemic illness may play a role in the psychosis disease process. Consequently, since it is debatable whether to treat young people at risk of psychosis with antipsychotic drugs [154], therapy with omega-3 fatty acids and nutraceuticals, having no clinically noteworthy negative side effects, can be discussed as a possible initial treatment for these subjects.

Eighteen trials evaluating omega-3 fatty acids administration in UHR subjects [28,29,30,31,32], patients with first-episode psychosis [33,34,35,36,37,40,43], and stable schizophrenia patients [45,47,48,50,51] have been performed over the past decade.

Five of these studies evaluated the administration of EPA (between 700 and 840 mg) and DHA (between 480 and 560 mg) in UHR subjects [28,29,30,31,32]. The results showed that omega-3 fatty acids improved inflammatory and blood profiles [28,31] and reduced the risk of progression to psychotic disorder [29]. In a post hoc study [29], they also showed that most individuals in the omega-3 fatty acids group did not show severe functional deterioration and did not experience attenuated psychotic symptoms at follow-up. On the contrary, two studies in which omega-3 fatty acids were administered in a UHR cohort showed no clinical benefits [30,32]. In a RCT [30], omega-3 fatty acids plus cognitive-behavioral case management (CBCM) or placebo plus CBCM were administered in a wide sample of young subjects at risk of developing psychosis. The authors reported an improvement in transition rates to psychosis with no significant differences between the two groups. A possible explanation could be that amelioration is obtained because of the CBCM that both groups received and that this could have hidden the effects of omega-3 fatty acids. Some authors have [32] showed a predominantly anti-inflammatory action of omega-3 fatty acids on plasma status in UHR subjects, but this effect did not appear to induce clinical benefits at 6- and 12-month follow-up.

Regarding the first episode of psychosis and omega-3 fatty acids administration, we found eight RCTs [33,34,35,36,37,40,43]. Emsley’s trial administered fatty acids as monotherapy, with no antipsychotic support [33], while trials performed by Pawelczyk and colleagues [34,35,36,37,41] tested EPA and DHA in adjunction to antipsychotics. The duration of these trials ranged from 26 weeks to 2 years. PUFA dosages ranged between 2.2 and 3 g/day. In the study [33], omega-3 fatty acids were administered to prevent relapse during antipsychotic discontinuation in remitted first-episode psychosis and did not produce significant benefits for symptom severity and functioning or relapse rate after antipsychotic discontinuation. Pawełczyk’s studies reported promising data on EPA and DHA in first-episode psychosis in terms of symptom reduction and neurobiological changes. The findings showed an improvement in general, psychotic, negative, and depressive symptoms [34,35], a decrease in the oxidative stress status of plasma with a positive effect on global and negative symptoms [35], and an increase in telomerase levels in peripheral blood cells with a positive effect on depressive symptoms and severity of illness [36,37,41].

It has beendemonstrated, with the MATRICS Consensus Cognitive Battery (MCCB) and the Brief Psychiatric Rating Scale (BPRS), that higher levels of omega-3 fatty acids were significantly correlated with better social cognition, while higher levels of arachidonic acid (an omega-6 fatty acid) were significantly correlated with hostility/non-cooperation [40]. In addition, patients treated with risperidone associated with omega-3 fatty acids (EPA + DHA) for 16 weeks had a significant longitudinal improvement in social cognition. The authors, therefore, provided new evidence on the differential role of omega-3 versus omega-6 fatty acids in treating the symptoms and neuropsychological deficits of recent-onset psychosis. Furthermore, it was shown that omega-3 supplementation can determine white matter MRI changes in patients with recent-onset psychosis after risperidone treatment [43].

Five RCTs were performed in patients with stable schizophrenia and omega-3 fatty acid supplementation [45,47,48,50,51]. Four studies showed a positive effect of omega-3 fatty acids on the symptom domains of schizophrenia [45,47,48,51]. The duration of the studies ranged from 8 to 16 weeks. Daily doses of PUFAs were between 0.9 and 1.4 g. EPA was found superior to placebo and DHA in reducing the psychotic symptoms [45], depressive symptoms [48], and anxious symptoms [48] of schizophrenia. One of these studies indicated that omega-3 fatty acids were useful in reducing violent behaviors, but these patients had no improvement in the positive or negative symptoms of schizophrenia [47]. In a RCT [50], it was found that the administration of omega-3 fatty acids for 12 weeks in patients with schizophrenia and metabolic syndrome improved triglyceride metabolism. Despite these encouraging initial data, it is hard to draw any conclusion on the medium- and long-term efficacy of omega-3 fatty acids in stable schizophrenia. Trials performed in the stable phase of illness have too short a duration to establish the long-lasting effects of these agents. It is interesting to note that the majority of studies found a positive effect of pure EPA or a fatty acid composition with predominantly EPA, at least when added to a stable antipsychotic treatment. Available data suggested that EPA or the composition of fatty acids with a high proportion of EPA could be more effective in the early periods of schizophrenia than in the chronic phase of the disorder. Studies using pure or predominant EPA in high-risk subjects are lacking and should be conducted. Tang et al. [51] showed that omega-3 fatty acids had beneficial effects on cognitive function in patients with metabolic syndrome, which is paralleled by enhanced brain-derived neurotrophic factor levels. These findings were consistent with the hypothesis that omega-3 fatty acid metabolism is implicated in the etiology of negative symptoms of schizophrenia [146] and with the notion that oxidative damage to lipids is connected to the process of neuroprogression and the expression of negative symptoms [155].

Regarding the administration of probiotics, the literature is unfortunately scarce: there are no trials in which probiotics have been administered in UHR subjects. Huang et al., in their two trials on patients receiving olanzapine therapy at the first psychotic episode, showed that administering probiotics (*Bifidobacteri*, *Lactobacilli*, *Enterococci*) improved the metabolic profile of the patient and that the concomitant administration of dietary fiber helps prevent the weight gain expected when administering antipsychotic therapies such as olanzapine [44]. The other three studies on the effect of probiotics [49,53,55] showed an improvement in PANSS score [49,53] and metabolic profile [49,53,55]. In addition to probiotics, some of these studies included the concomitant administration of vitamin D [49], selenium [53], and prebiotics [55].

There are three studies investigating vitamin D supplementation [42,49,57]. No studies have been performed on UHR subjects. The randomized study by Ghaderi et al. [49], which has already been mentioned, showed an improvement in the PANSS score and metabolic profile, but this investigation included the co-administration of probiotics, so the result may not be conclusive for vitamin D. A study [42] examined adults aged 18 to 65 years within 3 years of first presentation of a functional psychotic disorder and showed no significant differences between those who received vitamin D supplementation and those receiving placebo. The study by Kalejahi et al. [57] was conducted in patients suffering from schizophrenia and hypovitaminosis D and highlighted improvements regarding the level of GSK-3 β (an important biomarker in schizophrenia) and insulin resistance. 

A study [38] evaluated whether vitamins B6 and B12 and folic acid can lower the level of homocysteine (which is elevated in patients affected by schizophrenia and correlates with illness severity) and improve symptomatology and neurocognition in the first episode of psychosis. It showed that vitamin B supplementation for 12 weeks did not improve overall psychopathology and global neurocognition, but had specific neuroprotective properties in attention/vigilance, particularly in patients with elevated homocysteine levels, patients with affective psychosis, and female patients.

Some RCTs have been performed on molecules that prevent oxidative stress. This process and the consequent impairment of parvalbumin oligodendrocytes and interneurons may underlie alterations in brain connectivity in schizophrenia. In addition, the level of the brain antioxidant glutathione in the medial prefrontal cortex was positively related to better functional connectivity along the cingulum bundle in healthy controls, but not in patients with initial psychosis. Three RCT studies have been conducted on alpha lipoic acid (ALA, an agent with antioxidative properties) with divergent results [46,54,56]. The dosage of ALA was between 100 and 300 mg/day. Sanders’ study showed improvement in the Brief Rating Scale scores, neurocognitive parameters, and extrapyramidal symptoms and a reduction in lipid peroxidation [46]. Mishra’s study showed an improvement in the Scale for the Assessment of Negative Symptoms (SANS), but not in the Scale for the Assessment of Positive Symptoms (SAPS). Therefore, it demonstrated improvement only in negative symptoms of psychosis [54]. On the contrary, De Lima’s study found no differences between those taking ALA and the placebo group [56].

A randomized controlled study was carried out with N-acetyl-cysteine, a precursor of glutathione, administered for 6 months. It was found that functional connectivity increased along the cingulate and, more precisely, between the anterior caudal part and the isthmus of the cingulate cortex. Consequently, this study suggests that increasing glutathione levels in the brain through N-acetyl-cysteine supplementation may improve functional connectivity in the brain [39].

Maguire et al. tried to administer the coenzyme Q10 at a dosage of 300 mg/day for 6 months, but there were no cognitive, psychological, or health-related benefits in patients with schizophrenia and schizoaffective disorder. The study had important limitations, including poor adherence, small sample size, and attrition, that likely reduced the effect estimated. So, findings should be considered preliminary [52]. 

The results of the RCTs are displayed in Table 1.

### 4.2. Autism Spectrum Disorders

Autism spectrum disorder (ASD) is a complex developmental disease that begins in infancy or earlier and lasts throughout the individual’s lifetime. It is characterized by stereotyped behavior and deficits in social communication, interaction, and perception [156,157]. There is no practical and targeted treatment for ASD, which has become a major worldwide health problem [158]. In fact, current treatment encompasses mainly education and rehabilitation interventions, without significant improvement in the core symptoms. The etiology and mechanisms of ASD are not yet completely understood. Many studies suggested a possible link between ASD and multiple environmental as well as genetic risk factors [159]. Moreover, increasing evidence supports the hypothesis that children who suffer from autism are more likely to experience inconvenience related to the gastrointestinal tract (GIT), including food allergies, dysbiosis, inflammatory bowel disease, and indigestion [160]. Gastrointestinal disturbances are commonly encountered comorbidities that are thought to be not only another symptom of ASD but also to play an active role in modulating the expression of social and behavioral symptoms. Therefore, nutritional interventions are used by patients with ASD to alleviate gastrointestinal and behavioral symptoms, but there is no consensus regarding optimal nutritional therapy [161].

There is a general agreement on the significant role of omega-3 fatty acid metabolism in neurodevelopmental disorders and symptom improvement. Even in ASD, as in other psychiatric disorders, low plasma levels of omega-3 fatty acids have been found [162,163].

Eight studies have been identified exploring the effect of omega-3 fatty acid supplementation in ASD [58,59,60,61,66,68,75,78]. Among them, four reported an improvement in ASD symptoms [85,86,87,88]. In these studies, 722 mg to 1.3 g of omega-3 fatty acids/day were administered in rather wide cohorts of children. The duration of trials varied from 6 weeks to 12 months. They showed improvement in hyperactivity [58], irritability [68], stereotyped behaviors, social communication, and Gilliam Autism Rating Scale (GARS) [75]. In the study performed by Ooi et al. [61], post-treatment, blood fatty acid levels were significantly correlated with changes in the core symptoms of ASD, and baseline levels of blood fatty acids were also predictive of response to the omega-3 fatty acid treatment. Moreover, ASD symptoms were positively correlated with cytokine and chemokine levels in the bloodstream and cerebral spinal fluid [164]. Two trials tested fatty acid supplementation and examined inflammatory markers for changes in behavior [60,68]. Mankad et al. suggested worsened externalizing behaviors with increasing levels of IL-10 and IL-1β but no clear path connecting supplementation to changes in cytokines and to subsequent behavior [60]. Similarly, in another study they [68] tested interactions between baseline IL-1β levels and treatment assignment but did not measure changes in inflammation due to treatment. The mechanisms by which fatty acids might improve ASD symptoms are not well understood, which led us to hypothesize that omega-3 fatty acid supplementation would interrupt detrimental neurological pathways by reducing inflammation. Keim et al. [78] evaluated the effect of omega-3 and omega-6 fatty acid supplementation for 90 days in children (ages 2 < 6 years) recently diagnosed with ASD. The authors found that treatment increased omega-3 and omega-6 fatty acid levels (1.40 mol% for EPA and 1.62 mol% for DHA) and reduced IL-2 levels compared to placebo (−0.17 pg/mL, 95% CI −0.31, −0.02, d = −0.62).

Discordant results were reported by three other placebo-controlled studies [91,92,93] that showed no significant differences between treated patients and controls in autism symptoms. These studies were conducted in smaller cohorts of children, with a daily omega-3 fatty acid dosage of 0.75 g to 1.5 g for a period of 2–6 months. In two RCTs [60,66], the administration of EPA + DHA was envisaged, while in another trial [59], the administration of 0.2 g/day of DHA was provided in monotherapy.

Parellada and colleagues [66] investigated omega-3 fatty acid supplementation in ASD for 8 weeks (962 mg/d and 1155 mg/d for children and adolescents, respectively). Treatment with omega-3 fatty acids improved the erythrocyte membrane omega-6/omega-3 ratio in comparison to the placebo group. Nevertheless, the authors did not find a significant difference in behavioral measures (Social Motivation and Social Communication subscale scores) between groups.

A growing number of studies suggested the importance of probiotics in improving the balance of the gut microbiota and therefore the symptoms of ASD. For this reason, some studies have been carried out in which probiotics were administered [62,63,69,70,74,79]. In these RCTs, several nutraceuticals were administered, at different doses, including stumps of *Lactobacillus*, *Bilidumbacteria*, and *Streptococcus*. The observation period ranged from 1 to 12 months. These studies stated that intake of probiotics resulted in a better Bacteroidetes/Firmicutes ratio [62] and improvement of gastrointestinal symptoms [63,70], autism symptoms [63,70,74], disruptive and rule-breaking behaviors [69], hyperactivity/impulsivity [69], adaptive behaviors [79], and social preference [79]. In addition to probiotics, maltose [79], fructo-oligosaccharide [70], and oxytocin [74] were also administered in some studies.

A small open-label clinical trial evaluated the impact of microbiota transfer therapy (MTT) on gut microbiota composition and gastrointestinal and ASD symptoms of children diagnosed with ASD. MTT involved a 2-week antibiotic treatment, intestinal cleansing, and then extended fecal microbiota transplantation (FMT) using a high initial dose followed by daily and lower maintenance doses for 7–8 weeks. After MTT, ASD behavioral symptoms and gastrointestinal symptoms improved. The changes persisted for at least 8 weeks after the end of treatment, suggesting a long-term benefit [65].

Furthermore, integrative therapy with vitamin D has been attempted [68,71], in monotherapy [71] or in association with fatty acids [68], with a dosage range from 2000 IU/day to 6000 IU/day for a period of 12–15 weeks. In the study by Javadfar et al., the scores of the Childhood Autism Rating Scale (CARS) and the Autism Treatment Evaluation Checklist (ATEC) significantly improved, while the Adult Behavior Checklist (ABC-C) score and serum levels of serotonin and IL-6 were unchanged. Mazahery and collaborators [71] showed a reduction in irritability and hyperactivity, but it must be considered that in this study children received omega-3 fatty acids in association with vitamin D.

Since folate plays a key role in neural development during the embryonic and fetal period and in the first years of life [165,166], high-dose folinic acid was also taken into consideration and showed an improvement in verbal communication in children with ASD. Two recent studies [73,76] tested the effect of folinic acid supplementation in a sample of children with autism. The dosage of folinic acid ranged from 10 to 50 mg/day and the duration of the studies ranged from 10 to 12 weeks. It should be noted that in the study by Batebi et al. [76], folinic acid was administered concomitantly with the antipsychotic risperidone, while in another RCT [73], it was provided in monotherapy or without changes in therapeutic management in the 8 weeks preceding the study. The findings showed an improvement in the Autism Diagnostic Observation Schedule (ADOS) score [73], inappropriate speech, stereotypic behavior, and hyperactivity/noncompliance [76]. On the other hand, no differences were recorded regarding irritability and lethargy/social withdrawal [76].

Some evidence supported the hypothesis that children born extremely preterm had a higher risk of developing ASD and associated behaviors than children born at a later gestational age [167,168,169,170]. Four studies were conducted to assess the risk of developing ASD in children born prematurely [64,67,72,77]. Omega-3-6-9 fatty acids were administered at doses ranging between 0.5 g and 1 g. The follow-up period ranged from 13 to 26 weeks. These compounds showed a beneficial effect on early language development [64], interpersonal relationship adaptive behavior [77], and ASD symptoms measured with the Brief Infant Toddler Social Emotional Assessment (BITSEA) ASD scale [67]. On the contrary, the study by Boone et al. [72] performed in a larger cohort of children did not find significant differences between cases and controls.

Data obtained from studies of autism spectrum disorders are summarized in Table 2.

### 4.3. Major Depression

Major depressive disorder (MDD) is an episodic and recurrent disorder characterized by evident depression of mood and loss of interest, as well as significant deterioration of cognitive abilities and autonomic functions, lasting more than two weeks, but usually several months. There is commonly remission between two episodes. The treatment of major depressive episodes is mainly based on monoaminergic medications. However, despite the presence of several antidepressant drugs, their effectiveness in some patients is still partial. A promising intervention to improve antidepressant treatment could be the use of adjunctive nutraceuticals which are now considered a versatile, tolerable, and effective adjunctive treatment to reduce the impact of depressive symptoms and improve the functioning of MDD patients [171].

Some investigations reported that patients with MDD have a lower level of EPA and DHA in their peripheral tissues (plasma, serum, and red blood cells) than control subjects [172,173]. A dietary intake of omega-3 fatty acids could be linked to a decreased risk of MDD [173,174,175,176,177] and improvement in white matter integrity [178]. Moreover, the anti-inflammatory properties of omega-3 fatty acids, particularly EPA, could be crucial to preventing the onset of depression [179]. The available evidence suggests that depression rates are rising among children and adolescents, potentially driven by alterations in environmental factors [180]. One of these factors could be the change in eating habits, with the consumption of high-energy and nutrient-poor foods, as well as the abandonment of traditional diets that included a greater intake of plant foods and quality proteins [181]. This change has resulted in an increased consumption of omega-6 and a depletion of omega-3 fatty acids [182], which leads to an imbalance of fatty acid composition in plasma and erythrocytes, with a negative impact on central nervous system neuronal membranes and serotonin transport. Available data suggested a relationship between low levels of EPA and DHA and depressive symptoms in adulthood [173,183,184]. Therefore, EPA and DHA can exert antidepressant, anti-inflammatory, and neuroprotective properties, although the exact molecular mechanism underlying their effects is not yet completely clear.

In the last decade, eleven RCTs have been carried out on the effects of omega-3 fatty acids in treating major depression [80,81,82,83,84,85,86,88,90,96,104]. Fatty acids were administered both in monotherapy and as supplementation to ongoing pharmacotherapy or psychotherapy. The majority of studies tested the efficacy of the combination of EPA and DHA. Doses ranged from 0.2 to 4 g/day of EPA and from 0.3 to 1.4 g/day of DHA. To assess the level of depressive and depression-related symptoms, rather heterogeneous evaluation instruments were used (Hamilton Depression Rating Scale, Montgomery–Asberg Depression Rating Scale, Beck Depression Inventory, Childhood Depression Rating Scale, Childhood Depression Inventory, Edinburgh Postnatal Depression Scale, Geriatric Depression Scale, Hopkins Symptom Checklist Depression Scale, Postpartum Depression Screening Scale).

Three studies tested the combination of EPA and DHA in improving depressive symptoms in adolescents. Young et al. studied the benefits of 2 g/day of omega-3 fatty acid supplementation combined with individual–family psychoeducational psychotherapy (PEP) in comparison with PEP monotherapy, omega-3 fatty acid monotherapy, or placebo [84]. The authors showed that combined therapy could reduce behavioral symptoms, such as hyperactivity, impulsivity, and opposition that often appear with depression in young people. Similar results were obtained by Trebatická and colleagues [96] who enrolled depressed adolescents who were randomized to receive 2.4 g/day omega-3 (including 1 g EPA + 0.75 g DHA) or 2.467 g/day omega-6 (linoleic acid). They found a significant reduction in Children’s Depression Inventory (CDI) scores in the group receiving omega-3 [96]. Less encouraging findings were collected by Gabbay and collaborators [85] in patients who received EPA + DHA (2:1 ratio) versus placebo. The authors concluded that omega-3 fatty acids did not appear superior to placebo in adolescents with MDD.

Five studies [80,83,86,90,104] that evaluated the supplementation of omega-3 in adult patients suffering from unipolar depression showed that EPA and DHA were effective in reducing depressive symptoms [80,90], that subjects with MDD and a high number of inflammatory biomarkers had a better response to EPA compared to placebo and a lower response to DHA compared to placebo [83], that omega-3 fatty acid supplementation was superior to placebo in improving anxiety, sleep, and emotion regulation [86], and that 4 g/day of EPA can alleviate MDD in overweight individuals with elevated inflammatory markers [104]. On the other hand, three studies with similar characteristics [81,82,88] did not obtain statistically significant results in favor of omega-3 fatty acid intake.

Although some studies have yielded non-significant or unfavorable results, there is widespread agreement in considering omega-3 fatty acids as promising agents that can ameliorate depressive symptoms in MDD in combination with antidepressants or even in monotherapy [185,186]. A focal point to be considered is the difference in efficacy of the two fatty acids more commonly tested: EPA and DHA. Available evidence indicated that pure or predominant EPA, but not pure DHA, is effective in major depression [187].

To propose increasingly comprehensive treatments for major depression that also include new therapeutic agents, the neuromodulatory effects of the microbiome and its role in depression, anxiety, and stress responses have acquired great interest. In particular, the gut microbiota is suspected to affect brain functions and behavior through the gut–brain axis [13,188]. In the last ten years, thirteen trials have been performed to evaluate the impact of probiotics on depressive disorder [89,91,92,95,97,98,102,103,106,107,108,109,110]. Eleven studies showed encouraging results. Kazemi et al. [92] compared the effects of probiotics (*Lactobacillus helveticus* and *Bifidobacterium longum*) or galactooligosaccharides (GOS) with placebo and reported benefits in patients who received probiotics in terms of reduction of depressive symptoms (measured with BDI), while no significant effect was registered in the group that received GOS. Improvement of depressive symptoms in patients who received probiotics has also been found in other RCTs [91,102,103,106,109,110]. Moreover, probiotics also alleviated gastrointestinal symptoms [91], reduced IL6 blood levels [98], improved sleep quality [102,103,110], and reduced anxious symptoms [103,109,110]. Two RCTs [95,107] evaluated the efficacy of the probiotic *Lactobacillus plantarum HEAL9* and of *Lactobacillus helveticus Rosell^®^-52* plus *Bifidobacterium longum Rosell^®^-175* with the addition of 200 mg/day of S-adenosylmethionine (SAMe). The authors found that these compounds improved depressive symptoms evaluated with the Zung Self-Rating Depression Scale (Z-SDS) [95], the Patient Health Questionnaire-9 (PHQ-9), and the HDRS (Hamilton Depression Rating Scale) [107]. Depressive symptoms also improved in patients with subthreshold depression [107]. Since the gut–brain microbiota axis is linked to depression and cognition, Schneider et al. investigated the effect of high-dose probiotic supplementation on the cognitive symptoms related to depression and highlighted the potential of microbiota-related regimens to treat cognitive symptoms of depression [108]. Less promising findings were reported in two other studies [89,97], which found no differences in cognitive functions between the group receiving probiotics and the control group.

Eight studies were conducted on vitamin D [87,93,94,99,100,101,105]. Low vitamin D levels seemed to be associated with a dysregulated hypothalamic–pituitary–adrenal (HPA) axis and depression. In many studies, the increased intake of vitamin D is reflected in increased serum concentration [93,94,99,101,105], but this is not always the case. In some studies, Vitamin D serum concentration did not significantly increase after supplementation [87,93,100]. This is probably due to the low dose of Vitamin D that was administered. RCTs that evaluated whether the increase in serum level of Vitamin D may improve psychiatric symptoms reported a reduction of depressive [94,99,105] and anxious symptoms [100].

Data of studies of MDD are summarized in Table 3.

### 4.4. Bipolar Disorder

Bipolar disorders (BDs) include bipolar I disorder and bipolar II disorder. They are characterized by recurrent episodes of major depression and hypomania or mania. In particular, the diagnosis of bipolar I disorder requires at least one manic episode that may have been preceded and may be followed by a hypomanic or major depressive episode. Bipolar II disorder is characterized by the lifetime experience of at least one episode of major depression and at least one hypomanic episode. Bipolar depressive episodes are approximately like major depressive episodes. Manic and hypomanic episodes are characterized by a marked change in mood (abnormally elevated, expansive, or irritable mood) and behavior during discrete periods. There are frequently and for varying lengths of time free gaps in between episodes. The most common initial presentation is depression and the age of onset is between 15 and 25 years. Early diagnosis and treatment are associated with a more favorable prognosis [189]. A growing number of investigations reported that inflammatory mechanisms may be considered mediators of pathophysiology in BDs [11,190] and that the omega-3/omega-6 fatty acid ratio, implicated in processes of inflammation, is often unbalanced in patients with BDs.

A cross-sectional study conducted by McNamara and collaborators [191] observed that the increased risk of developing bipolar disorder was associated with deficits in EPA and DHA levels in erythrocytes. The authors stated that low levels of omega-3 fatty acids can be considered a biomarker of prodromal risk for BD in young subjects. In light of this evidence, three recent studies, in which omega-3 was administered to young people at risk of developing bipolar disorder, found increased levels of omega-3 fatty acids in erythrocytes [120,126] and decreased functional connectivity of the amygdala with the right inferior temporal gyrus. These changes were associated with the reduction of the Childhood Depression Rating Scale–Revised score and the Clinical Global Impression-Severity Scale score [126]. 

To our knowledge, eight studies testing the effects of omega-3 fatty acids in patients with BD are available in the chosen time frame [112,113,118,122,124,127,129,130]. Daily dosages of omega-3 fatty acids ranged from approximately 650 mg to 2.25 g. One RCT suggested that the combined treatment with omega-3 fatty acids and inositol reduced symptoms of mania and depression in prepuberal children with mild to moderate bipolar spectrum disorders [113]. Another study suggested that the supplementation of omega-3 fatty acids improved mood variability, energy, irritability, and pain (measured using the Ecological Momentary Assessment scale) and reduced serum concentrations of inflammatory factors such as tumor necrosis factor (TNF-α), Interleukiyn-6 (IL-6), and highly sensitive C Reactive Protein (hs-CRP) [129]. Additionally, it was observed that omega-3 fatty acid intake can prevent relapse of bipolar depression [130] and reduce the severity of depression [129,130]. On the other hand, two studies did not confirm these promising data. In the study performed by Mcphilemy et al. [122], in which 1 g of EPA plus 1 g of DHA was administered for 52 weeks in comparison with placebo, no differences were registered in the relapse rate of mood episodes and in the time to relapse. Moreover, the HDRS, the Clinical Global Impression (CGI), and the Global Assessment of Functioning (GAF) scores did not show significant changes [122]. Similarly, the RCT published by Saunders et al. [127] found no significant differences in the severity of mood symptoms between those who consumed a diet high in omega-3 (1.5 g/day) and low in omega-6 and a control diet standardized to the usual American distribution of 150 mg/day of omega-3 and a higher omega-6 content [127].

Three studies investigated the efficacy of the association of omega-3 fatty acids with psychotherapeutic interventions. Fristad and colleagues [112] compared the combination of psychoeducational intervention and 2000 mg/day of omega-3 fatty acids with placebo and active monitoring in youths with subsyndromal BD (BD not otherwise specified, cyclothymic disorder). Combined therapy was associated with greater improvement in depressive symptoms, but not in manic symptoms. However, all participants experienced and reported a decline in manic symptoms over the course of the study. The same research group [124] performed a follow-up study of 2–5 years after participation in the previous RCT to evaluate the long-lasting effects of combined therapy. The authors found that in participants, regardless of treatment group, manic symptom severity, executive functioning, and global functioning remained comparable to the end of the RCT. In addition, they found that those who persisted in supplementation with omega-3 fatty acids (it is not specified how long they did) had lower depressive symptom severity than those who discontinued treatment. The third study [118] aimed to assess the impact of 1870 mg/day of omega-3 fatty acid supplementation as a single treatment or in association with psychoeducational therapy on executive functions in youths with mood disorders (depressive disorder, cyclothymic disorder, or bipolar disorder not otherwise specified). Subjects receiving omega-3 fatty acid supplementation had a significant improvement in executive functions over time and the majority of patients reported concurrent improvements in dysphoric mood, irritability, and self-esteem [118].

Based on the promising results of probiotics on unipolar depression, their administration was also attempted in two studies including patients with bipolar disorders [116,125]. In a study dispensed *Lactobacillus rhamnosus strain GG* and *Bifidobacterium animalis* subsp. *lactis strain Bb12* in patients discharged after hospitalization for mania for 24 weeks. Probiotic supplementation was found to be associated with a lower rate of re-hospitalization [116]. In the study conducted by Sabouri et al. [125], BD symptoms were not investigated, but no changes in markers of blood inflammation and oxidative stress were observed after 8 weeks of probiotic therapy in patients with BD type 1 [125].

Three studies on vitamin supplementation were conducted in the period examined [114,123,131]. As vitamin D supplementation seems to improve symptoms of unipolar depression, Marsh et al. [114] administered 5000 IU/day of vitamin D for 12 weeks to patients suffering from BD and hypovitaminosis D, but did not find improvements in depressive, anxious, and manic symptoms measured with the Montgomery–Åsberg Depression Rating Scale (MADRS), the Hamilton Anxiety Rating Scale (HAM-A), and the Young Mania Rating Scale (YMRS). Furthermore, as vitamin B6, in addition to having an anti-inflammatory effect and reducing homocysteine, plays a role in serotonin and dopamine regulation, two studies were conducted on this agent. In the first study, [123] vitamin B6 (80 mg) was administered daily in BD patients during a manic episode with psychotic manifestations in association with lithium. No significant differences in mood were observed in patients compared to controls. On the contrary, in the RCT by Zandifar et al. [131] 66 patients were randomized to take, in addition to standard lithium treatment, 100 mg/day of vitamin B1, 40 mg/day of vitamin B6, or placebo, and it was found that vitamin B6, but not vitamin B1, improved mood compared to placebo and that both improved quality of sleep compared to placebo [131].

Extensive research in the pathophysiology of BD points to the existence of mitochondrial and bioenergetic dysfunction. So, the implementation of nutraceuticals with mitochondrial activity has received growing interest in the last few years. Three studies were focused on testing the effects of the mitochondrial modulators in BD: creatine monohydrate, coenzyme Q10, and N-acetylcysteine [117,119,121]. In an RCT with a placebo, creatine monohydrate was tested as an adjunctive therapy to the usual treatment for bipolar depression, with no significant effects on symptoms of the disorder [119]. Coenzyme Q10 (CoQ10) (a mitochondrial modulator, antioxidant, and anti-inflammatory agent) was found to be more efficacious in reducing depressive symptoms in patients with bipolar depression than placebo [117]. N-acetylcysteine (NAC), mitochondrial-enhancing nutraceuticals, or placebo were administered in a rather wide sample of patients suffering from BD. The authors observed that the participants with a better diet quality including N-acetylcysteine (Australian Recommended Food Score) reported a major decrease in subjective depressive symptoms and a greater clinician-rated improvement irrespective of treatment and time [121]. 

Given the potential effect of folic acid as an adjunctive therapy in major depression, this compound was also tested in one RCT in patients with BD. In the RCT by Sharpley et al. [111], folic acid (2.5 mg/day) or placebo was administered to boys at familial risk of developing BD. The authors demonstrated that the difference in the incidence of mood disorders in the two groups was not significant. However, in a post hoc analysis of this study examining the 18 participants who reached the primary endpoint, the median time to the onset of the mood disorder was 5 months in the placebo group and 15.5 months in the folate group. In a more recent open-label clinical study [115], L-methylfolate in combination with usual treatment was found to be efficacious in reducing symptoms of depression in BD, but controlled studies are needed to confirm this initial evidence.

Data of studies of BD are summarized in Table 4.

### 4.5. Personality Disorders

The effect of omega-3 fatty acids and nutraceuticals in personality disorders has mainly been tested in patients with borderline personality disorder (BPD) and, unfortunately, there is no recent literature on this topic. 

BPD is a widespread, long-lasting mental disorder characterized by impulsive-behavioral dyscontrol, unstable affective states, distorted self-image, and dysfunctional relationships. 

The hybrid dimensional-categorical model of the DSM-5 describes personality disorders in terms of five personality domains: negative affectivity, antagonism, disinhibition, detachment, and psychoticism. In particular, BPD is diagnosed when traits belonging to the three domains of negative affectivity, antagonism, and disinhibition are present [192].

As several investigations showed a positive effect of omega-3 fatty acids on impulsive and aggressive symptoms in healthy and psychiatric subjects (suffering from Attention Deficit Hyperactivity Disorder, autism spectrum disorder, and bipolar disorder) [193,194,195], the efficacy of supplementation of these agents has also been tested in patients with BPD, who often show impulsive-behavioral dyscontrol and aggressive conducts.

To our knowledge, in the last ten years, there is only one RCT, lasting 12 weeks, in which 1.2 g/day of EPA and 0.6 g/day DHA were administered concomitantly with the usual valproic acid-based therapy or valproic acid as monotherapy in 43 patients with BPD. The study shows that the combined therapy improves symptoms, in particular regarding impulsive symptoms, behavioral dyscontrol, anger, and self-mutilating behavior [132]. Furthermore, there is a 6-month follow-up study of this RCT showing that improvement persists regarding anger outbursts [133].

In recent decades, it was found that omega-3 fatty acids were effective in reducing aggression and symptoms of depression when used without additional medications [196]. More recent studies suggested that these compounds were useful when used in combination with conventional pharmacotherapies to reduce depressive symptoms, impulsivity, self-harm, and anger outbursts [132,133].

Data of studies on BPD are summarized in Table 5.

The Cochrane systematic review focused on the pharmacological treatment of borderline personality disorder [197] suggested that, in addition to antipsychotics, mood stabilizers, and antidepressants, omega-3 fatty acids showed effects on anger, brief psychotic symptoms, and dissociative phenomena. However, a new edition of this review questioned the value of this evidence.

There are no data in the literature on other personality disorders, but one study showed that omega-3 fatty acid supplementation for a 6-month period is helpful in reducing aggressive and antisocial behaviors in a cohort of 324 children who had not received a specific psychiatric diagnosis, especially in females and those with psychopathic type personalities [134]. 

Regarding nutraceuticals, we found only scarce data on personality disorders or abnormal behaviors. A Korean study [15] found a close association between gut microbiota imbalance and facets of neuroticism (a heterogeneous trait consisting of multiple facets including anxiety, hostility, depression, self-consciousness, impulsivity, and vulnerability to stress). The study showed an inversely proportional correlation between anxiety vulnerability and the richness of the gut microbial flora: the greater these traits, the lower the richness of the microbiome. Furthermore, a significant difference was found between the low and high anxiety, self-awareness, impulsivity, and vulnerability groups. Specifically, patients with high anxiety and vulnerability had a low abundance of *Christensenellaceae* belonging to Firmicutes Clostridia, high self-awareness was correlated with a low abundance of *Alistipes* and *Sudoligranulum*, and impulsivity was correlated with a low abundance of *Oscillospirales*.

Some authors [13,198,199,200] highlighted a significant role in the etiology of BPD for early developmental processes, including prenatal stress and maternal dysbiosis, with effects mediated through the infant gut microbiome and its influence on amygdala development. The amygdala has an important role in the cortex and brain connectivity that may explain some of the abnormalities present in BPD, such as excessive activation of the amygdala towards negative emotions and reduced frontal regulation [201]. It is necessary to investigate whether the high levels of stress and dysphoria that are frequently associated with BPD mediate some of its consequences through gut dysbiosis and increased gut permeability.

Differences in the gut microbiome of BPD patients compared to controls were found in the Bacteroidetes/Firmicutes ratio (BFR), a marker widely used to detect alterations in gut microbial composition. In the sample, the BFR was higher in the BPD than in the control cohort, especially when controlled for BMI and depression. Furthermore, differences in the taxonomic composition of the gut microbiota were identified and revealed a potential dysbiosis among short-chain-fatty-acid-producing bacteria in BPD [202].

### 4.6. Reviews and Meta-Analysis

Over the last decade, several narrative reviews, systematic reviews, and meta-analyses have been carried out on nutraceuticals in psychiatric disorders. 

The collected data suggested that EPA more than fatty acid composition may be effective in the early stages of schizophrenia and the chronic stages of the disorder, but the number of studies is still insufficient. More studies with standardized outcome measures, longer durations, and follow-up periods should be performed, as well as in samples at high risk for psychosis [22,23,26,27].

The efficacy of omega-3 fatty acids in autism spectrum disorders was not confirmed. Available findings are conflicting in their conclusions. Some reviews stated that no significant differences in the severity of autism symptoms were observed after omega-3 fatty acid treatment [162,163], except for lethargy, stereotypies, and hyperactivity [200]. However, a combination of omega-3 fatty acids and vitamin D produced some good effects on the social and behavioral outcomes of these patients [162]. 

Some meta-analyses showed positive results for EPA and DHA in monotherapy [185,186] and in combination with other drugs [185,203], particularly as an adjunctive therapy to antidepressants for moderate to severe major depressive disorder [203]. Nevertheless, some authors stated that the quality of the evidence was not good enough to determine the effectiveness of omega-3 fatty acids in the treatment of DDM [204,205,206,207,208]. The results indicated the therapeutic potential of EPA in depression at proportions ≥ 60% of the total composition EPA + DHA and doses ≥ 1 g/day and <2 g/day [187]. There is no evidence that the depressive symptoms that often occur during menopause are alleviated by omega-3 fatty acids [209].

In the systematic review [210] on nutraceutical supplementation in BDs, it was found that the adjunction of unsaturated fatty acids (mainly omega-3), folic acid, zinc, and CoQ10-probiotic to pharmacotherapy improved mood-related symptoms, while non-significant effects emerged from creatine, carnitine, vitamin D, inositol, or NAC. 

Regarding personality disorders, a recent review [211] evaluated whether omega-3 fatty acids improved symptoms of borderline personality disorder. The most consistent results concerned affective dysregulation and impulsive behavioral dyscontrol. 

Reviews that considered the tolerability of omega-3 fatty acids were concordant in maintaining these agents as safe and well tolerated in all psychiatric disorders, except for some episodes of diarrhea or dysgeusia [212].

Considering that dysbiosis is frequently discovered in patients with psychiatric disorders, probiotics and prebiotics have received considerable attention as potential psychiatric therapies. A systematic review [213] argued that limited inferences can be made regarding the efficacy of probiotics in schizophrenia and that the clinical utility of probiotics in this disorder has yet to be validated by future clinical trials.

As regards ASD, the Authors stated that despite promising preclinical results, prebiotics and probiotics have shown overall limited efficacy in the management of behavioral symptoms in children with ASD and that studies with standardized strains and fixed durations are needed [213]. Another review [214] and one meta-analysis [215] proposed approximately the same conclusions: probiotics and prebiotics did not significantly improve the severity of gastrointestinal abnormalities and psychopathology in ASD. On the other hand, a review [216] suggested the presence of changes in ASD symptoms and gastrointestinal symptoms after intervention with prebiotics, probiotics, and transplantation of fecal microbiota. However, the results should be taken with caution because there are very few studies that analyze the efficacy of long-term treatments and compare the different combinations of agents.

Most of the literature is concordant in maintaining that probiotics may have an antidepressant and anxiolytic effect, but the pooled effects were reduced by the paucity of trials with clinical samples [217,218,219,220,221,222,223,224,225,226]. A very recent meta-analysis supported probiotic implementation in pregnancy and breastfeeding women in order to prevent depressive and anxious symptoms [227]. In contrast, some reviews and meta-analyses [228,229,230] claimed that probiotic supplementation had an overall insignificant effect on mood and that interstudy discrepancies concerning probiotic dosage, bacterial strains, and strain combinations limited the comparability of clinical studies. Furthermore, most randomized trials were conducted in healthy individuals, making it difficult to extend findings to depressed patients. 

The opportunity to explore the therapeutic potential of vitamin D supplementation in subjects with psychiatric disorders was sustained by several authors [231,232]. Neonatal vitamin D deficiency seems to be linked to an increased risk of schizophrenia. Also, patients with psychotic onset and stable schizophrenia have an increased risk of vitamin D deficiency compared to healthy controls [100,233], but these data have to be interpreted with caution as the reduced circulating vitamin D levels could be due to the poor general health and often unbalanced diet typical of patients with psychosis [233]. 

A meta-analysis [234] focused on maternal and neonatal vitamin D levels showed a trend of decreased early-life vitamin D concentration in patients with autism spectrum disorder and suggested that children with reduced maternal or neonatal vitamin D had a 54% higher likelihood of developing ASD. According to these analyses, vitamin D status could be related to the risk of ASD [234]. A maternal serum vitamin D level of more than 30 nmol/L was associated with lower odds of offspring with ASD [235]. Two reviews suggested that vitamin D supplementation is useful in MDD, especially in adults and in patients with a severe degree of depressive symptoms [236,237]. To our knowledge, there are no systematic reviews or meta-analyses about vitamin D supplementation in personality disorders.

The Canadian Network for Mood and Anxiety Treatment (CANMAT) guidelines (2022) concluded that among nutraceuticals with Grade A evidence, varying levels of support were found for adjunctive omega-3 fatty acids, vitamin D, adjunctive probiotics, adjunctive zinc, methylfolate, and adjunctive S-adenosylmethionine (SAMe) in the treatment of unipolar depression. Monotherapy with omega-3 fatty acids, folic acid, vitamin C, tryptophan, creatine, inositol, magnesium, NAC, and SAMe was not supported by sufficient evidence for this use. In bipolar disorder, omega-3 fatty acids had weak evidence of efficacy for bipolar depression, while NAC was not recommended. Vitamin D, NAC, and methylfolate were recommended to varying degrees in the treatment of the negative symptoms of schizophrenia, while omega-3 fatty acids were not, although evidence suggests a role for the prevention of transition to psychosis in high-risk youth with potential pre-existing fatty acid deficiency [238].

## 5. Conclusions

The present review investigates the use of omega-3 fatty acids and nutraceuticals in the treatment of psychiatric disorders, particularly schizophrenia, ASD, MDD, BD, and personality disorders. Our research predominantly focused on randomized controlled trials conducted in this field over the past decade.

Although the role of omega-3 fatty acids, particularly EPA and DHA, in psychiatric disorders has received growing interest in recent years and has been studied in an increasing number of clinical trials, there is a lack of general agreement on their efficacy and the available evidence is controversial and inconclusive. A major obstacle to drawing more definitive conclusions about the effects of these agents is the large heterogeneity among randomized trials. Differences in methods are notable and concern sample size, diagnostic criteria, type and doses of omega-3 fatty acids (e.g., EPA, DHA, or both, or the addition of omega-6, or omega-9 fatty acids), the association with standard drugs, duration of studies, and follow-up assessments.

The main evidence of the effectiveness of EPA and DHA was obtained in mood disorders. In particular, omega-3 supplementation has proven effective in reducing depressive symptoms in MDD and in BD, mostly in depressive but also in manic phases. Initial data on adolescent or older cohorts are promising, but further studies on larger samples are needed. Supplementation with a daily dose between 0.6 g and 4 g of omega-3 fatty acids has proven effective in reducing depressive symptoms in MDD and BD. We can also infer from follow-up studies, albeit sparse, that long-term treatments lead to more stable and persistent improvement in depressive symptoms.

Findings regarding schizophrenia and related psychotic disorders are still debated. In UHR subjects, the available evidence neither rejects nor supports the use of omega-3 fatty acids, while supplementation may be more effective in the early stages of schizophrenia and in the chronic stages of the disorder as an adjunct to antipsychotic treatment. It is possible to deduce that supplementation with a daily dose of 1–2 g of omega-3 fatty acids has a protective effect on the rate of conversion to psychosis and that omega-3 fatty acids have positive effects both on positive and negative symptoms of schizophrenia, as well as on the risk of relapse. 

In ASD, the number of clinical trials and reviews has increased since our previous review [22,23,27], but a consensus among researchers has not yet been reached. The most favorable results concern the effect of high-dose omega-3 fatty acids in terms of reduction of hyperactivity, improvement of lethargy, and development of social interactions in children with ASD. As the most recent studies did not show important differences between the treatment groups, omega-3 fatty acid integration can only be proposed as a complement to other therapies in this clinical population.

In borderline personality disorder, the supplementation of omega-3 fatty acids has obtained favorable results on some core symptoms: impulsiveness, self-harm, and anger. Developments in this field are promising, but are still limited. Moreover, there are no new RCTs on omega-3 fatty acids compared to our previous reviews [22,23].

Most of the studies examined in this review agree on the improvements that omega-3 fatty acid integration produce from an inflammatory and metabolic point of view, both for preventive and therapeutic purposes.

Although the results are not sufficiently consistent, the available results on the effects of omega-3 fatty acids in different psychiatric disorders are promising in terms of clinical efficacy and good tolerability. The lack of significant adverse effects is a reason to consider the potential role of these agents, especially in the treatment of young or elderly individuals. Further investigations are needed to make more specific clinical objectives clear, to define the most appropriate modes of administration (doses, duration of treatment, omega-3 fatty acid molecules, and compositions), and to provide reliable guidelines for the use of these agents in clinical practice.

A more recent and constantly growing field of research is that of probiotics. The microbiome could regulate the immune system, maintain intestinal and blood–brain barrier integrity, and modulate the parasympathetic nervous system, brain function, and neuroinflammation. Understanding the regulatory mechanisms that govern microbiome–central nervous system interaction may help in understanding the pathological mechanisms that underlie several psychiatric and neurodevelopmental disorders, including depression and ASD. Results from human studies have shown that probiotics and microbial transplants can positively influence anxiety, stress responses, and depression, and can therefore help patients suffering from various pathologies. 

There are no studies on probiotics in subjects at UHR for psychosis. Results about the efficacy of probiotics in the first psychotic episode are not conclusive, while in stable schizophrenia, they seem to be effective both from a symptomatic and a metabolic point of view. In ASD, studies show that probiotics improve both the characteristic symptoms of the disorder and the gastrointestinal dysfunctions. Regarding mood disorders, results are different in MDD and BD. In the majority of trials including patients with MDD, there was an improvement in depressive symptoms. In BD, a study showed a reduction in re-hospitalization rates after manic episodes, while other investigations found only poor improvement of symptoms.

Other molecules examined in this review have shown benefits, but their supplementation has not been tested in all disorders. For example, vitamin D showed some benefits in patients with schizophrenia and hypovitaminosis D; N-acetylcysteine was found to be efficacious in patients experiencing their first psychotic episode in terms of functional connectivity within the cingulate cortex; microbiota transfer therapy produced some effect in improving behavioral symptoms in ASD; folinic acid had unsatisfactory results in schizophrenia, while it appeared to improve symptoms of ASD; coenzyme Q10 had a beneficial effect in bipolar depression. It should also be noted that no studies on nutraceuticals are available in patients with personality disorders.

Designing and conducting such studies with higher levels of standardization and in larger cohorts represents a promising field of research that can be explored to help patients who do not respond to any of the traditional therapeutic regimens or who discontinued the usual therapies due to side effects or pharmaco-phobic traits.

## Figures and Tables

**Table 1:** Schizophrenia.

High-Risk Psychosis					
Study	Study Design	Drugs and Dose	Cohort	Treatment Duration	Results
Smesny et al., 2014 [28]	Randomized, double-blind, placebo-controlled trial	700 mg/day EPA + 480 mg/day DHA	80 (13 to 25 years)	12 weeks	Normalizing PLA2 activity and d-6-desaturase-mediated metabolism of omega-3 and omega-6
Amminger et al., 2015 [29]	Post hoc subgroup analysis (Amminger et al., 2010)	700 mg/day EPA + 480 mg/day DHA	81 (13 to 25 years)	12 weeks	Reduced risk of progression to psychotic disorder and psychiatric morbidity
McGorry et al., 2017 [30]	Randomized, double-blind, placebo-controlled trial	840 mg/day EPA + 560 mg/day DHA + CBCM	304 (13 to 40 years)	24 weeks	No differences
Alqarni et al., 2020 [31]	Randomized, double-blind, placebo-controlled, clinical replication trial (McGorry et al., 2017)	840 mg/day EPA + 560 mg/day DHA	304 (13 to 40 years)	24 weeks	Increase of level of omega-3 in erythrocyte
Susai et al., 2022 [32]	Randomized, clinical trial	840 mg/day EPA + 560 mg/day DHA	268 (18.47 ± 4.49 years)	52 weeks	Reduced inflammatory profile No clinical effects
**First-Episode Psychosis**					
**Study**	**Study Design**	**Drugs and Dose**	**Cohort**	**Treatment** **Duration**	**Results**
Emsley et al., 2014 [33]	Randomized, double-blind, placebo-controlled trial	2 g/day EPA + 1 g/day DHA + α-LA 300 mg/day	33 (18 to 48 years)	104 weeks	Relapse prevention of psychotic symptoms
Pawelzcyk et al., 2016 [34]	Randomized, double-blind, placebo-controlled trial	2.2 g/day omega-3 (EPA + DHA)	71 (16 to 35 years)	26 weeks	↓ psychotic symptoms ↓ depressive symptoms Increase in level of functioning
Pawelzcyk et al., 2017 [35]	Secondary outcome analysis of a randomized trial (Pawelzcyk et al., 2016)	2.2 g/day omega-3 (EPA + DHA)	71 (16 to 35 years)	26 weeks	↓ psychotic symptoms
Pawelzcyk et al., 2018 [36]	Secondary outcome analysis of a randomized trial (Pawelzcyk et al., 2016)	2.2 g/day omega-3 (EPA + DHA)	71 (16 to 35 years)	26 weeks	Increase of level of telomerase in peripheral blood cells ↓ depressive symptoms
Pawelzcyk et al., 2019 [37]	Secondary outcome analysis of a randomized trial (Pawelzcyk et al., 2016)	2.2 g/day omega-3 (EPA + DHA)	71 (16 to 35 years)	26 weeks	Increase of BDNF level ↓ depressive symptoms
Allott et al., 2019 [38]	Randomized, double-blind, placebo-controlled trial	5 mg/day folic acid + 0.4 mg/day vit. B12 + 50 mg/day vit. B6	120 (15 to 25 years)	12 weeks	Reduction of homocysteine levels neuroprotective in attention/ vigilance
Mullier et al., 2019 [39]	Pilot randomized, placebo-controlled trial	2700 mg/day N-acetyl-cysteine	20 (25 ± 6 years)	24 weeks	Increase of functional connectivity within the cingulate cortex
Szeszko et al., 2021 [40]	Randomized, double-blind, placebo-controlled trial	740 mg/day EPA + 400 mg/day DHA + risperidone (dosage not available)	50 (average age: 21.5 years)	16 weeks	Increase in social cognition
Pawelzcyk et al., 2021 [41]	Findings from a randomized controlled study (Pawelzcyk et al., 2016)	2.2 g/day omega-3 (EPA + DHA)	71 (16 to 35 years)	26 weeks	↓ psychotic symptoms Reduction of TG level ↓ MetS risk
Gaughran et al., 2021 [42]	Randomized, multisite, double-blind, placebo-controlled, parallel-group clinical trial	120,000 UI/month vit. D	149 (18 to 65 years)	24 weeks	No differences
Lyall et al., 2021 [43]	Randomized, double-blind, placebo-controlled trial	740 mg/day EPA + 400 mg/day DHA + risperidone or placebo + risperidone (dosage not available)	37 (MRI performed on 18) (average age: 21.8 years)	16 weeks	↓ MRI
Huang et al., 2022 [44]	Randomized clinical trials	≈ 5 × 10^7^ CFU/day probiotics (*Bifidobacteri*, *Lactobacilli*, *Enterococci*) + 15–20 mg/day olanzapine	90 (18 to 50 years)	12 weeks	↓ insulin resistance
Huang et al., 2022 [44]	Randomized clinical trials	≈ 5 × 10^7^ CFU/day probiotics (*Bifidobacteri*, *Lactobacilli*, *Enterococci*) + 20 g/day dietary fibers + 15–20 mg/day olanzapine	60 (18 to 50 years)	12 weeks	↓ metabolic profile
**Stable Schizophrenia**					
**Study**	**Study Design**	**Drugs and Dose**	**Cohort**	**Treatment** **Duration**	**Results**
Jamilian et al., 2014 [45]	Randomized, double-blind, placebo-controlled trial	1 g/day omega-3	60 (23 to 39 years)	8 weeks	↓ psychotic symptoms
Sanders et al., 2017 [46]	Open-label trial	100 mg/day ALA	10 (38.5 ± 7.26 years)	16 weeks	↓ Brief Psychiatric Rating Scale ↓ neurocognitive parameters ↓ extrapyramidal symptoms ↓ lipid peroxidation
Qiao et al., 2018 [47]	Randomized, double-blind, placebo-controlled trial	540 mg/day EPA + 360 mg/day DHA	50 (18 to 60 years)	12 weeks	↓ violence, but no improvement in positive and negative symptoms
Robinson et al., 2019 [48]	Randomized, placebo-controlled trial	EPA 740 mg + DHA 400 mg/day	50 (4 of them BD) (15 to 40 years)	16 weeks	↓ confusion, anxiety, depression, irritability, and tiredness/fatigue
Ghaderi et al., 2019 [49]	Randomized, double-blind, placebo-controlled trial	50,000 UI Vit. D/2 weeks + 8 × 10^9^ CFU/day probiotic (*L. acidophilus*, *B. bifidum*, *L. reuteri*, *L. fermentum*)	60 (25 to 65 years)	12 weeks	↓ psychotic symptoms ↓ metabolic profile
Xu et al., 2019 [50]	Randomized, double-blind, placebo-controlled trial	720 mg/day EPA + 480 mg/day DHA + olanzapine (dosage not available)	80 patients with schizophrenia + MetS (24 to 33 years)	12 weeks	↓ TG metabolism
Tang et al., 2020 [51]	Randomized, placebo-controlled trial	360 mg/day EPA +240 mg/day DHA + olanzapine (dosage not available)	80 (18 to 45 years)	12 weeks	Increase in cognitive function
Maguire et al., 2021 [52]	Randomized, placebo-controlled trial	300 mg/day Coenzyme Q10	72 (age not available)	24 weeks	No differences
Jamilian et al., 2021 [53]	Randomized, double-blind, placebo-controlled trial	8 × 10^9^ CFU/day probiotics (*L. acidophilus*, *B. lactis*, *B. bifidum*, *B. longum*) + 200 μg/day selenium	60 (18 to 60 years)	12 weeks	↓ psychotic symptoms ↓ metabolic profile
Mishra et al., 2022 [54]	Randomized, double-blind, placebo-controlled trial	300 mg/day ALA	20 (18 to 65 years)	8 weeks	↓ positive symptoms
Sevillano-Jiménez et al., 2022 [55]	Randomized clinical, double-blind, balanced-block	Probiotic + prebiotics (individual program)	50 (18 to 65 years)	26 weeks	↓ MetS
De Lima jr et al., 2023 [56]	Randomized, double-blind, placebo-controlled study	100 mg/day ALA	Not available	16 weeks	no differences
Kalejahi et al., 2023 [57]	Randomized, controlled trial	2000 UI/day vit. D	48 (schizophrenia + hypovitaminosis D) (18 to 65 years)	8 weeks	↓ waist circumference ↓ psychotic symptoms Reduction of GSK-3β level ↓ metabolic profile

Abbreviations: EPA = eicosapentaenoic acid; DHA = docosahexaenoic acid; ↓ = improvement of; α-LA = alpha lipoic acid; ALA = alpha lipoic acid; TG = triglycerides, MetS = metabolic syndrome; GSK-3β = glycogen synthase kinase 3 beta; PLA2 = phospholipase 2A; CBCM = cognitive-behavioral case management; BDNF = Brain-Derived Neurotrophic Factor; Vit. = vitamin; L. = Lactobacillus, B. = Bifidobacterium; BD = Bipolar Disorder; MRI = Magnetic Resonance Imaging; CFU = colony-forming unit.

**Table 2:** Autism Spectrum Disorder.

Autism Spectrum Disorder					
Study	Study Design	Drugs and Dose	Cohort	Treatment Duration	Results
Bent et al., 2014 [58]	Randomized, controlled trial	1.3 g/day of omega-3 (and 1.1 g of EPA+ DHA)	57 children (5 to 8 years)	6 weeks	↓ hyperactivity
Voigt et al., 2014 [59]	Randomized, double-blind, placebo-controlled trial	0.2 g/day DHA	48 children (3 to 10 years)	26 weeks	No differences
Mankad et al., 2015 [60]	Randomized, placebo-controlled trial	0.75–1.5 g/day EPA + DHA	38 children (2 to 5 years)	26 weeks	No differences
Ooi et al., 2015 [61]	Open label trial	192 mg/day EPA + 840 mg/day DHA	41 patients (7 to 18 years)	12 weeks	Improve SRS-2 ↓ Attention Problems Syndrome Scales of CBCL
Tomova et al., 2015 [62]	Pilot study	3 capsules of probiotics (Children Dophilus^®^: 3 stumps *Lactobacilli* 60% + 2 stumps *Bifidumbacteria* 25% + 1 stump *Streptococci* 15%)	29 children (10 ASD children, their 9 non-ASD siblings, 10 non-ASD children) (2 to 17 years)	16 weeks	↓ Bacteroidetes/Firmicutes ratio Increase of *Lactobacillus* spp.
Grossi et al., 2016 [63]	Case study	9 × 10⁹ CFU/day *Bifidobacteria* + 8 × 10^10^ CFU/day *Lactobacilli* + 20 × 10^10^ CFU/day *Streptococci*	A 12-year-old child	4 weeks	↓ GI symptoms ↓ ASD symptoms ↓ ADOS-2 score
Sheppard et al., 2017 [64]	Pilot randomized, controlled trial	338 mg EPA + 225 mg DHA + 83 mg GLA + 306 mg Omega 9	31 children (18–38 months of age born at ≤29 weeks of gestation)	12 weeks	↓ early language development in children at risk for ASD
Dae-Wook Kang et al., 2017 [65]	Open-label study	MTT treatment protocol (antibiotic + bowel cleanse + FMT)	38 children (7 to 17 years)	18 weeks (8 weeks follow up)	↓ GI symptoms ↓ behavioral ASD symptoms
Parellada et al., 2017 [66]	Randomized, crossover, placebo-controlled trial	962 mg/day omega-3 for children or 1155 mg/day omega-3 for adolescents	68 patients (5 to 17 years)	8 weeks	No differences
Keim et al., 2018 [67]	Randomized, double-blind, placebo-controlled trial	338 mg/day EPA + 225 mg/day DHA + 83 mg/day GLA	31 patients (18–38 months of age who were born at ≤29 weeks of gestation)	12 weeks	↓ ASD symptoms
Mazahery et al., 2019 [68]	Randomized, controlled trial	2000 UI/day Vit. D or 722 mg/day DHA or 2000 UI/day vit. D + 722 mg/day DHA	117 (2.5 to 8 years)	52 weeks	Vit. D and omega-3: ↓ irritability vit. D: ↓ hyperactivity
Liu et al., 2019 [69]	Randomized, double-blind, placebo-controlled Trial	3 × 10^10^ CFU/capsule/day (*L. plantarum PS128*)	80 (7 to 15 years)	4 weeks	↓ disruptive and rule-breaking behaviors ↓ hyperactivity/impulsivity
Wang et al., 2020 [70]	Controlled, clinical trial	10^10^ CFU/pack/day probiotics (*B. infantis*, *L. Rhamnosus*, *B. lactis*, *L. paracasei*) + FOS	26 (3 to 9 years)	12 months	↓ severity of autism ↓ GI symptoms
Javadfar et al., 2020 [71]	Randomized, clinical trial	300 UI/kg (max 6000 UI/day) vit. D	43 (8.41 ± 2.87 years)	15 weeks	Improved CARS Improved ATEC
Boone et al., 2020 [72]	Secondary analysis of a randomized trial not available	200 mg/day DHA + 200 mg/day AA	377 (10–16 months of age born at ≤35 weeks of gestation)	26 weeks	No differences (caregiver reported)
Renard et al., 2020 [73]	Randomized, placebo-controlled trial	10 mg/day folinic acid	19 children (3 to 10 years)	12 weeks	Improved ADOS score
Kong et al., 2021 [74]	Randomized, double-blinded, placebo-controlled pilot trial	6 × 10 ^10^ CFU/day (*L. plantarum PS128*) + oxytocin (dosage not available)	35 (3 to 20 years)	28 weeks	↓ ABC ↓ SRS ↓ CGI
Doaei et al., 2021 [75]	Randomized, clinical trial	1 g/day omega-3	54 children (5 to 15 years)	8 weeks	↓ stereotyped behaviors improve social communication ↓ GARS score
Batebi et al., 2021 [76]	Randomized, double-blind, placebo-controlled trial	2 mg/kg (up to 50 mg)/day folinic acid + risperidone (initiating dose of 0.5 mg/day with a dose increase of 0.5 mg per week, maximum 1.5 mg/day)	55 children (4 to 12 years)	10 weeks	↓ inappropriate speech ↓ stereotypic behavior ↓ hyperactivity/noncompliance
Boone et al., 2022 [77]	Secondary analysis of a randomized clinical trial not available	338 mg EPA + 225 mg DHA + 83 mg GLA + 280 mg omega-6 + 306 mg omega-9	31 children (18–38 months of age born at ≤29 weeks of gestation)	12 weeks	↓ depressive behavior ↓ internalizing behavior ↓ interpersonal relationship adaptive behavior
Keim et al., 2022 [78]	Randomized, double-blind, controlled trial	112 mg EPA+ 67 mg DHA+ 122 mg omega-6 (included 32 mg GLA) + 83 mg omega-9	72 (2 to 6 years)	12 weeks	Reduction of IL2 level
Schmitt et al., 2023 [79]	Randomized, controlled trial	2 × 10 ^10^ CFU *L. Reuteri* + 200 mg Sepadex^®^ (dextran microparticles) + 74 mM maltose/day	15 (15 to 45 years)	4 weeks	↓ adaptive behavior ↓ social preference

Abbreviations: EPA = eicosapentaenoic acid; DHA = docosahexaenoic acid; Vit. D = vitamin D; AA = arachidonic acid; GI = gastrointestinal; FOS = fructo-oligosaccharide; IL = interleukin; GLA = gamma-linolenic-acid; FMT = fecal microbiota transplant; GARS = Gilliam Autism Rating Scale-second edition; MTT = Microbiota transfer therapy; SRS-2 = Social Responsiveness Scale Improve Social; CBCL = Child Behavior Checklist; ASD = autism spectrum disorder; CFU = colony-forming unit; L. = lactobacillus; B. = bifidobacterium; CARS = Childhood Autism Rating Scale; ATEC = Autism Treatment Evaluation Checklist; CGI = Clinical Global Impression; ABC = Autistic Behavior Checklist; ADOS = Autism Diagnostic Observation Schedule; ↓ = improvement of.

**Table 3:** Major Depression Disorder.

Major Depression Disorder					
Study	Study Design	Drugs and Dose	Cohort	Treatment Duration	Results
Ginty et al., 2015 [80]	Preliminary randomized and placebo-controlled trial	1.4 g/day omega-3 (EPA + DHA) monotherapy	23 (18 to 21 years)	3 weeks	↓ BDI scores over time
Mischoulon et al., 2015 [81]	Randomized, double-blind, placebo-controlled trial	1 g/day EPA or 1 g/day DHA or placebo	196 (age not available)	8 weeks	No differences
Park et al., 2015 [82]	Randomized, double-blind, placebo-controlled trial	1140 g/day EPA + 0.6 g/day DHA + standard therapy	35 (18 to 65 years)	12 weeks	No differences
Rapaport et al., 2016 [83]	Proof-of-concept study	1060 mg/day EPA + 260 mg DHA or 180 mg EPA + 900 mg/day DHA or placebo	155 (18 to 80 years)	8 weeks	Subjects with MDD and a high number of inflammatory biomarkers had a better response to EPA than the placebo and a lower response to DHA than the placebo
Young et al., 2017 [84]	Randomized, placebo-controlled trial	1.4 g/day EPA + 0.2 g/day DHA + 0.4 g/day other omega-3 + IF-PEP	72 (7 to 14 years)	12 weeks	↓ co-occurring behavior symptoms
Gabbay et al., 2018 [85]	Double-blind, placebo-controlled trial	2:1 ratio of EPA to DHA: Initial dose of 1.2 g/day. Doses were raised in increments of 0.6 g/day every 2 weeks (maximum possible dose of 3.6 g/day, combined 2.4 g EPA + 1.2 g DHA)	51 psychotropic medication-free adolescents with MDD (12 to 19 years)	10 weeks	No differences
Jahangard et al., 2018 [86]	Randomized, double-blind, placebo-controlled trial	1000 mg/day omega-3 + 50–200 mg/day sertraline	50 (18 to 65 years)	12 weeks	↓ depression, anxiety, sleep, and patients’ competencies to regulate their emotions
Hansen et al., 2019 [87]	Randomised, multicenter, double-blind, placebo-controlled trial	2800 UI /day Vit. D	62 (18 to 65 years)	12 weeks (+12 weeks follow up)	No differences
Tayama et al., 2019 [88]	Randomized, double-blind, placebo-controlled trial	1000 mg/day EPA + 500 mg/day DHA	20 (18 to 75 years)	12 weeks	No differences
Chahwan et al., 2019 [89]	Randomized, triple-blind, placebo-controlled trial	Ecologic^®^Barrier (*B. bifidum W23*, *B. lactis W51*, *B. lactis W52*, *L. acidophilus W37*, *L. brevis W63*, *L. casei W56*, *L. salivarius W24*, *L. lactis W19 and L. lactis W58* (total cell count 1 × 10^10^ CFU/day)	71 (23 to 48 years)	8 weeks	No differences
Parletta et al., 2019 [90]	Randomized, placebo-controlled trial	200 mg/day EPA + 900 mg/day DHA + Mediterranean-style diet	152 (18 to 65 years)	26 weeks	↓ depressive symptoms ↓ mental health
Karakula-Juchnowicz et al., 2019 [91]	Double-blind, placebo-controlled clinical study protocol	3 × 10^9^ CFU *L. helveticus Rosell^®^-52* + *B. longum*	120 (18 to 60 years)	12 weeks	↓ GI symptoms ↓ depressive symptoms
Kazemi et al., 2019 [92]	Randomized, controlled trial	*L. helveticus + B. longum* (probiotic) or galactooligosaccharides (prebiotics) or placebo	110 (36.5 ± 8.03 years)	8 weeks	Probiotics: ↓ BDI Prebiotics: no differences
De Koning et al., 2019 [93]	Randomized, placebo-controlled trial	1200 UI/day Vit. D	155 (60 to 80 years)	52 weeks	Increase of Vit. D serum level No clinical differences
Alavi et al., 2019 [94]	Randomized clinical trial	50,000 IU/week Vit. D	78 older adults aged over 60 years	8 weeks	Increase of Vit. D serum level ↓ GDS-15
Saccarello et al., 2020 [95]	Randomized, double-blind, placebo-controlled Study	200 mg/day SAMe + 1 × 10⁹ CFU/day *L. plantarum HEAL9*	90 (18 to 60 years)	6 weeks	↓ Z-SDS
Trebatickà et al., 2020 [96]	Randomized, double-blind, placebo-controlled trial	2.4 g/day omega-3 (including 1 g EPA + 0.75 g DHA) or 2.467 g/day omega 6 (linoleic acid)	60 children suffering from depressive disorder or mixed anxiety and depressive disorder (7 to 18 years)	12 weeks	↓ CDI score ↓ omega 6/omega-3
Reininghaus et al., 2020 [97]	Randomized, placebo-controlled trial	*B. bifidum W23 + B. lactis W51 + B. lactis W52 + L. acidophilus W22 + L. casei W56 + L. paracasei W20 + L. plantarum W62 + L. salivarius W24 + L. lactis W19* daily	82 (18 to 75 years)	4 weeks	No differences
Reiter et al., 2020 [98]	Monocentric, randomized, placebo-controlled trial	*B. bifidum W23 + B. lactis W51+ B. lactis W52 + L. acidophilus W22 + L. casei W56 + L. paracasei W20 + L. plantarum W62 + L. salivarius W24 + L. lactis W19* daily	61 (18 to 75 years)	4 weeks	Reduction of IL6 level
Kaviani et al., 2020 [99]	Randomized, double-blind, placebo-controlled trial	50,000 IU/ 2 weeks vit. D	56 (43 ± 11.15 years)	8 weeks	↓ depressive symptoms Increase of serum vit. D level
Zhu et al., 2020 [100]	Randomized, placebo-controlled trial	1600 mg/day Vit D	158 with hypovitaminosis D (18 to 60 years)	26 weeks	No differences in depression symptoms; improved anxiety symptoms
Libuda et al., 2020 [101]	Randomized, placebo-controlled trial	2640 UI vit. D/day	113 with hypovitaminosis D (18 to 60 years)	4 weeks	Increase of serum vit. D level ↓ DISYPS
Ho et al., 2021 [102]	Randomized, double-blind, placebo-controlled pilot trial	2 capsules (3 × 10^10^ CFU) *L. Plantarum PS128*	40 non-depressed patients with insomnia (20 to 40 years)	4 weeks	↓ BDI ↓ awakenings during the deep sleep stage
Joo Lee et al., 2021 [103]	Randomized, double-blind, placebo-controlled Trial	5 × 10^9^ CFU probiotics (4.0 × 10^9^ CFU for *L. reuteri NK33* + 1 × 10^9^ CFU for *B. adolescentis NK98*)	156 healthy adults with subclinical symptoms of depression, anxiety, and insomnia (19 to 65 years)	8 weeks	↓ quality of sleep ↓ IL-6 ↓ depressive symptoms at 4 and 8 weeks of treatment ↓ anxiety symptoms at 4 weeks
Mischoulon et al., 2022 [104]	Randomized, dose-finding clinical trial	1 g/day or 2 g/day or 4 g/day EPA	61 (age not available)	12 weeks	4 g/day EPA: ↓ depressive symptoms ↓ hs-CRP
Kaviani et al., 2022 [105]	Randomized, double-blind, placebo-controlled trial	50,000 IU cholecalciferol/2 weeks-1	56 (18 to 60 years)	8 weeks	Increase of Vit. D serum level ↓ depressive symptoms
Schaub et al., 2022 [106]	Randomized, placebo-controlled trial	900 billion CFU/day (*S. thermophilus + B. breve + B. longum + B. infantis + L. acidophilus + L. plantarum + L. paracasei + L. delbrueckii subsp. Bulgaricus*) + treatment-as-usual	47 (over 18 years)	4 weeks	↓ depressive symptoms
Ullah et al., 2022 [107]	Monocentric, randomized, cross-over, double-blind, placebo-controlled clinical trial	200 mg/day SAMe + 3 × 10^9^ CFU/day *L. helveticus Rosell^®^-52 + B. longum Rosell^®^-175*	80 patients with SD or MDD (18 to 65 years)	12 weeks	↓ SD symptoms ↓ MDD symptoms
Schneider et al., 2023 [108]	Secondary analysis of a randomized, placebo-controlled trial	*Bifidobacteria* 9 × 10^10^ CFU/g + *Lactobacilli* 8 × 10^10^ + *S. salivarius subsp. Thermophilus* 20 × 10^10^ resulting in a daily dose of 900 billion CFU/d + usual depression treatment	60 (over 18 years)	4 weeks	↓ cognitive function (verbal episodic memory and working memory)
Nikolova et al., 2023 [109]	Single-center, double-blind, placebo-controlled pilot randomized clinical trial	2 × 10^9^ CFU *B. subtilis*, *B. bifidum*, *B. breve*, *B. infantis*, *B. longum*, *L. acidophilus*, *L. delbrueckii subsp bulgaricus*, *L. casei*, *L. plantarum*, *L. rhamnosus*, *L. helveticus*, *L. salivarius*, *L. lactis and S. thermophilus*	49 MDD taking antidepressant medication, but having an incomplete response were studied (18 to 55 years)	8 weeks	↓ depressive symptoms ↓ anxiety symptoms
Zhu et al., 2023 [110]	Randomized, placebo-controlled trial	*L. plantarum JYLP-326* 2 vv/day	60 anxious 22-year-old students	3 weeks	↓ depression ↓ anxiety ↓ insomnia

Abbreviations: EPA = eicosapentaenoic acid; DHA = docosahexaenoic acid; MDD = Major depressive disorder; BDI = Beck Depression Inventory; GI = Gastrointestinal; CFU = colony forming units; HDRS = Hamilton depression rating scale; SAMe = S-adenosylmethionine; Z-SDS = Zung Self-Rating Depression Scale; SD = Subthreshold depression; GDS-15 = Geriatric Depression Scale-15; DISYPS-II = diagnostic system for mental disorders in childhood and adolescents, self-hand parent rating; CDI = Children’s Depression Inventory; IF-PEP = individual–family psychoeducational psychotherapy; B. = bifidobacterium or Bacillus; L. = Lactobacillus or Lactococco; S. = Streptococcus; Vit. = vitamin; IL = interleukin; ↓ = improvement of; hs-CRP = hs-C reactive protein.

**Table 4:** Bipolar Disorder.

		Bipolar Disorder			
Study	Study Design	Drugs and Dose	Cohort	Treatment Duration	Results
Sharpley et al., 2014 [111]	Randomized, double-blind, placebo-controlled trial	2.5 mg/day folic acid	112 with familial risk of mood disorder (14 to 24 years)	156 weeks	No differences
Fristad et al., 2015 [112]	Randomized, placebo-controlled trial	2000 mg/day omega-3 (including 1400 mg EPA + 200 mg DHA) and IF-PEP vs. AM using a 2 × 2 design	23 (7 to 14 years)	12 weeks	Manic symptoms improved over time without significant treatment effects Effect of IF-PEP on child depression compared with AM was medium to large Effect of omega-3 on depression was medium
Wozniak et al., 2016 [113]	Pilot study	1650 mg/day EPA + DHA + 2000 mg inositol or 1650 mg/day EPA + DHA + placebo or 2000 mg inositol + placebo	24 (5 to 12 years)	12 weeks	Omega-3 + inositol: ↓ symptoms of mania and depression
Marsh et al., 2017 [114]	Randomized, double-blind, placebo-controlled trial	5000 UI/day Vit. D	33 patients with vit. D deficiency (18 to 70 years)	12 weeks	No differences in depressive symptoms
Nierenberg et al., 2017 [115]	Open trial proof-of-concept registry	15 mg/day L-methyl folate	10 (18 to 75 years)	6 weeks	↓ MADRS ↓ Cohen’s d ↓ YMRS
Dickerson et al., 2018 [116]	Randomized, parallel two-group, placebo-controlled trial	*L. rhamnosus strain GG* + *B. animalis subsp. Lactis strain Bb12* (dosage not available)	66 patients who have been recently discharged following hospitalization for mania (18 to 65 years)	24 weeks	↓ rehospitalization
Mehrpooya et al., 2018 [117]	Double-blind placebo-controlled trial	200 mg/day Coenzyme Q10	69 (18 to 65 years)	8 weeks	↓ depressive symptoms
Vesco et al., 2018 [118]	Randomized controlled trial	1.87 g/day omega-3 or PEP or PEP + omega-3	95 (7 to 14 years)	12 weeks	Omega-3: ↓ executive functions ↓ dysphoric mood ↓ irritability ↓ self-esteem
Toniolo et al., 2018 [119]	Double-blind, placebo-controlled trial	6 g/day creatine monohydrate	35 (18 to 59 years)	6 weeks	No differences
McNamara et al., 2020 [120]	Placebo-controlled proton magnetic resonance spectroscopy trial	2130 mg/day omega-3 (EPA + DHA)	42 children with depressive symptoms with at least one parent with DB (9 to 21 years)	12 weeks	No clinical differences Increase of erythrocyte EPA + DHA levels
Ashton et al., 2020 [121]	Sub-study, randomized, placebo-controlled trial	N-acetyl-cysteine or mitochondrial-enhancing nutraceuticals (including N-acetyl-cysteine)	133 (21.3 to 72 years)	16 weeks	Better diet quality (irrespective of treatment and time): ↓ general depression and bipolar depression symptoms Greater clinician-rated improvement
McPhilemy et al., 2021 [122]	Randomized, placebo-controlled trial	1 g/day EPA+ 1 g/day DHA	80 (over 18 years)	52 weeks	No differences
Badrfam et al., 2021 [123]	Randomized, double-blind, placebo-controlled trial	80 mg/day vit. B6 + lithium (gradually increased dose to a therapeutic level of 0.8–1.2)	50 (18 to 65 years)	8 weeks	No differences
Fristad et al., 2021 [124]	Naturalistic follow-up study	2000 mg/day omega-3 (including 1400 mg EPA + 200 mg DHA) and IF-PEP vs. AM using a 2 · 2 design	38 (11 to 19 years)	104–260 weeks	↓ depressive symptoms ↓ youth emotion regulation skills and family communication
Sabouri et al., 2022 [125]	Randomized, double-blind, placebo-controlled trial	Probiotics	38 (age not available)	8 weeks	No differences in markers of inflammation and oxidative stress
McNamara et al., 2022 [126]	Placebo-controlled trial	2130 mg/day omega-3 (EPA + DHA)	39 depressed youth at high risk for developing BD type I (9 to 21 years)	12 weeks	↓ functional amygdala–right inferior temporal gyrus connectivity ↓ depressive symptoms Increase of erythrocyte EPA + DHA levels
Saunders et al., 2022 [127]	Randomized, parallel-group, modified double-blind, controlled	1.5 g omega-3 (EPA + DHA) + low omega-6 vs. control diet standardized (150 mg omega-3 + omega-6)	82 (over 18 years)	48 weeks (4-8-12 weeks of diet exposure)	No differences
Wozniak et al., 2022 [128]	Randomized, double-blind, placebo-controlled trial	1650 mg/day EPA + DHA + 2000 mg inositol or 1650 mg/day EPA + DHA + placebo or 2000 mg inositol + placebo	69 (5 to 12 years)	12 weeks	↓ YMRS (inositol + omega-3) ↓ HDRS (inositol + omega-3) ↓ antimanic and antidepressant effects
Eslahi et al., 2023 [129]	Randomized, double-blind, placebo-controlled trial	2 g/day omega-3 (including 180 mg EPA + 120 mg DHA)	60 (16 to 60 years)	8 weeks	↓ depression score ↓ TNF-α ↓ IL-6 ↓ hs-CRP
Zailani et al., 2024 [130]	Pilot randomized, placebo-controlled trial	420 mg/day EPA + 220 mg/day DHA + 0.2 mg/day tertiary-butylhydroquinone + 2.0 mg/day vit. E	31 (18 to 65 years)	26 weeks	↓ recurrence of bipolar depression ↓ depressive symptoms
Zandifar et., 2024 [131]	Randomized, placebo-controlled trial	100 mg/day vit. B1 or 40 mg/day vit. B6 or placebo + 900–1200 mg lithium	66 (18 to 65 years)	8 weeks	B6: ↓ symptoms during a manic episode + ↓ sleep status B1: no mood improvement, ↓ sleep status

Abbreviations: EPA = Eicosapentaenoic acid; DHA = Docosaesaenoic acid; IL-6 = interleukin 6; YMRS = Young Mania Rating Scale; HDRS = Hamilton Depression Rating Scale; Vit. = vitamin; CFU = colony-forming unit; L. = lactobacillus; B. = bifidobacterium; ↓ = improvement, IF-PEP = individual–family psychoeducational psychotherapy; AM = active monitoring; MADRS = Montgomery–Asberg Depression Rating Scale; DPA = n-3 docosapentaenoic acid; BD = Bipolar Disorder; hs-CRP = hs-C reactive protein; TNF = tumor necrosis factor.

**Table 5:** Borderline Personality Disorder.

Borderline Personality Disorder					
Study	Study Design	Drugs and Dose	Cohort	Treatment duration	Results
Bellino et al., 2014 [132]	Randomized, controlled trial	1.2 g/day EPA + 0.6 g/day DHA + 800–1300 mg/day valproic acid vs. 800–1300 mg/day valproic acid (plasma range: 50–100 μg/mL)	43 BPD patients (18 to 50 years)	12 weeks	↓ severity of BPDSI ↓ impulsive behavioral dyscontrol ↓ anger ↓ self-mutilating conduct
Bozzatello et al., 2018 [133]	Follow-up study to Bellino et al., 2014	1.2 g/day EPA + 0.6 g/day DHA + 800–1300 mg/day valproic acid vs. 800–1300 mg/day valproic acid (plasma range: 50–100 μg/mL)	34 patients with BPD (18 to 50 years)	24 weeks	↓ outbursts of anger
Raine et al., 2021 [134]	Randomized, double-blind, placebo-controlled Trial	300 mg DHA + 300 mg EPA + 180 mg alpha-linolenic acid + 60 mg DPA	324 children (11.89 years (SD 2.59))	52 weeks	↓ aggression, ↓ antisocial behavior

Abbreviations: EPA = eicosapentaenoic acid; DHA = docosahexaenoic acid; DPA = n-3 docosapentaenoic acid; BPDSI = borderline personality disorder severity index; ↓ = improvement of.

## References

[1] Lee S., Gura K.M., Kim S., Arsenault D.A., Bistrian B.R., Puder M. (2006). Current Clinical Applications of Ω-6 and Ω-3 Fatty Acids. Nutr. Clin. Pract..

[2] Sinn N., Milte C., Howe P.R.C. (2010). Oiling the Brain: A Review of Randomized Controlled Trials of Omega-3 Fatty Acids in Psychopathology across the Lifespan. Nutrients.

[3] Pusceddu M.M., Nolan Y.M., Green H.F., Robertson R.C., Stanton C., Kelly P., Cryan J.F., Dinan T.G. (2016). The Omega-3 Polyunsaturated Fatty Acid Docosahexaenoic Acid (DHA) Reverses Corticosterone-Induced Changes in Cortical Neurons. Int. J. Neuropsychopharmacol..

[4] Calder P.C., Bosco N., Bourdet-Sicard R., Capuron L., Delzenne N., Doré J., Franceschi C., Lehtinen M.J., Recker T., Salvioli S. (2017). Health Relevance of the Modification of Low Grade Inflammation in Ageing (Inflammageing) and the Role of Nutrition. Ageing Res. Rev..

[5] Zou R., El Marroun H., Voortman T., Hillegers M., White T., Tiemeier H. (2021). Maternal Polyunsaturated Fatty Acids during Pregnancy and Offspring Brain Development in Childhood. Am. J. Clin. Nutr..

[6] Ruiz-León A.M., Lapuente M., Estruch R., Casas R. (2019). Clinical Advances in Immunonutrition and Atherosclerosis: A Review. Front. Immunol..

[7] de la Presa Owens S., Innis S.M. (1999). Docosahexaenoic and Arachidonic Acid Prevent a Decrease in Dopaminergic and Serotoninergic Neurotransmitters in Frontal Cortex Caused by a Linoleic and Alpha-Linolenic Acid Deficient Diet in Formula-Fed Piglets. J. Nutr..

[8] Bozzatello P., Brignolo E., De Grandi E., Bellino S. (2016). Supplementation with Omega-3 Fatty Acids in Psychiatric Disorders: A Review of Literature Data. J. Clin. Med..

[9] Simopoulos A.P. (1999). Essential Fatty Acids in Health and Chronic Disease. Am. J. Clin. Nutr..

[10] Ergas D., Eilat E., Mendlovic S., Sthoeger Z.M. (2002). N-3 Fatty Acids and the Immune System in Autoimmunity. Isr. Med. Assoc. J..

[11] Saunders E.F.H., Ramsden C.E., Sherazy M.S., Gelenberg A.J., Davis J.M., Rapoport S.I. (2016). Omega-3 and Omega-6 Polyunsaturated Fatty Acids in Bipolar Disorder: A Review of Biomarker and Treatment Studies. J. Clin. Psychiatry.

[12] Mischoulon D., Freeman M.P. (2013). Omega-3 Fatty Acids in Psychiatry. Psychiatr. Clin. N. Am..

[13] Farooq R.K., Alamoudi W., Alhibshi A., Rehman S., Sharma A.R., Abdulla F.A. (2022). Varied Composition and Underlying Mechanisms of Gut Microbiome in Neuroinflammation. Microorganisms.

[14] Cryan J.F., O’Mahony S.M. (2011). The Microbiome-Gut-Brain Axis: From Bowel to Behavior. Neurogastroenterol. Motil..

[15] Park E., Yun K.E., Kim M.-H., Kim J., Chang Y., Ryu S., Kim H.-L., Kim H.-N., Jung S.-C. (2021). Correlation between Gut Microbiota and Six Facets of Neuroticism in Korean Adults. J. Pers. Med..

[16] Foster J.A., Rinaman L., Cryan J.F. (2017). Stress & the Gut-Brain Axis: Regulation by the Microbiome. Neurobiol. Stress.

[17] Singhal G., Jaehne E.J., Corrigan F., Toben C., Baune B.T. (2014). Inflammasomes in Neuroinflammation and Changes in Brain Function: A Focused Review. Front. Neurosci..

[18] Skaper S.D., Facci L., Zusso M., Giusti P. (2018). An Inflammation-Centric View of Neurological Disease: Beyond the Neuron. Front. Cell. Neurosci..

[19] Hallahan B., Garland M.R. (2005). Essential Fatty Acids and Mental Health. Br. J. Psychiatry.

[20] Gören J.L., Tewksbury A.T. (2011). The Use of Omega-3 Fatty Acids in Mental Illness. J. Pharm. Pract..

[21] Cooper R.E., Tye C., Kuntsi J., Vassos E., Asherson P. (2015). Omega-3 Polyunsaturated Fatty Acid Supplementation and Cognition: A Systematic Review and Meta-Analysis. J. Psychopharmacol..

[22] Bozzatello P., Rocca P., Mantelli E., Bellino S. (2019). Polyunsaturated Fatty Acids: What Is Their Role in Treatment of Psychiatric Disorders?. Int. J. Mol. Sci..

[23] Bozzatello P., Blua C., Rocca P., Bellino S. (2021). Mental Health in Childhood and Adolescence: The Role of Polyunsaturated Fatty Acids. Biomedicines.

[24] Brainard J.S., Jimoh O.F., Deane K.H.O., Biswas P., Donaldson D., Maas K., Abdelhamid A.S., Hooper L., PUFAH group (2020). Omega-3, Omega-6, and Polyunsaturated Fat for Cognition: Systematic Review and Meta-Analysis of Randomized Trials. J. Am. Med. Dir. Assoc..

[25] Kraguljac N.V., Montori V.M., Pavuluri M., Chai H.S., Wilson B.S., Unal S.S. (2009). Efficacy of Omega-3 Fatty Acids in Mood Disorders—A Systematic Review and Metaanalysis. Psychopharmacol. Bull..

[26] Agostoni C., Nobile M., Ciappolino V., Delvecchio G., Tesei A., Turolo S., Crippa A., Mazzocchi A., Altamura C.A., Brambilla P. (2017). The Role of Omega-3 Fatty Acids in Developmental Psychopathology: A Systematic Review on Early Psychosis, Autism, and ADHD. Int. J. Mol. Sci..

[27] Bozzatello P., De Rosa M.L., Rocca P., Bellino S. (2020). Effects of Omega-3 Fatty Acids on Main Dimensions of Psychopathology. Int. J. Mol. Sci..

[28] Smesny S., Milleit B., Hipler U.-C., Milleit C., Schäfer M.R., Klier C.M., Holub M., Holzer I., Berger G.E., Otto M. (2014). Omega-3 Fatty Acid Supplementation Changes Intracellular Phospholipase A2 Activity and Membrane Fatty Acid Profiles in Individuals at Ultra-High Risk for Psychosis. Mol. Psychiatry.

[29] Amminger G.P., Mechelli A., Rice S., Kim S.W., Klier C.M., McNamara R.K., Berk M., McGorry P.D., Schäfer M.R. (2015). Predictors of Treatment Response in Young People at Ultra-High Risk for Psychosis Who Received Long-Chain Omega-3 Fatty Acids. Transl. Psychiatry.

[30] McGorry P.D., Nelson B., Markulev C., Yuen H.P., Schäfer M.R., Mossaheb N., Schlögelhofer M., Smesny S., Hickie I.B., Berger G.E. (2017). Effect of ω-3 Polyunsaturated Fatty Acids in Young People at Ultrahigh Risk for Psychotic Disorders: The NEURAPRO Randomized Clinical Trial. JAMA Psychiatry.

[31] Alqarni A., Mitchell T.W., McGorry P.D., Nelson B., Markulev C., Yuen H.P., Schäfer M.R., Berger M., Mossaheb N., Schlögelhofer M. (2020). Supplementation with the Omega-3 Long Chain Polyunsaturated Fatty Acids: Changes in the Concentrations of Omega-3 Index, Fatty Acids and Molecular Phospholipids of People at Ultra High Risk of Developing Psychosis. Schizophr. Res..

[32] Susai S.R., Mongan D., Healy C., Cannon M., Nelson B., Markulev C., Schäfer M.R., Berger M., Mossaheb N., Schlögelhofer M. (2022). The Association of Plasma Inflammatory Markers with Omega-3 Fatty Acids and Their Mediating Role in Psychotic Symptoms and Functioning: An Analysis of the NEURAPRO Clinical Trial. Brain Behav. Immun..

[33] Emsley R., Chiliza B., Asmal L., du Plessis S., Phahladira L., van Niekerk E., van Rensburg S.J., Harvey B.H. (2014). A Randomized, Controlled Trial of Omega-3 Fatty Acids plus an Antioxidant for Relapse Prevention after Antipsychotic Discontinuation in First-Episode Schizophrenia. Schizophr. Res..

[34] Pawełczyk T., Grancow-Grabka M., Kotlicka-Antczak M., Trafalska E., Pawełczyk A. (2016). A Randomized Controlled Study of the Efficacy of Six-Month Supplementation with Concentrated Fish Oil Rich in Omega-3 Polyunsaturated Fatty Acids in First Episode Schizophrenia. J. Psychiatr. Res..

[35] Pawełczyk T., Grancow-Grabka M., Trafalska E., Szemraj J., Pawełczyk A. (2017). Oxidative Stress Reduction Related to the Efficacy of N-3 Polyunsaturated Fatty Acids in First Episode Schizophrenia: Secondary Outcome Analysis of the OFFER Randomized Trial. Prostaglandins Leukot Essent Fat. Acids.

[36] Pawełczyk T., Grancow-Grabka M., Trafalska E., Szemraj J., Żurner N., Pawełczyk A. (2018). Telomerase Level Increase Is Related to N-3 Polyunsaturated Fatty Acid Efficacy in First Episode Schizophrenia: Secondary Outcome Analysis of the OFFER Randomized Clinical Trial. Prog. Neuro-Psychopharmacol. Biol. Psychiatry.

[37] Pawełczyk T., Grancow-Grabka M., Trafalska E., Szemraj J., Żurner N., Pawełczyk A. (2019). An Increase in Plasma Brain Derived Neurotrophic Factor Levels Is Related to N-3 Polyunsaturated Fatty Acid Efficacy in First Episode Schizophrenia: Secondary Outcome Analysis of the OFFER Randomized Clinical Trial. Psychopharmacology.

[38] Allott K., McGorry P.D., Yuen H.P., Firth J., Proffitt T.-M., Berger G., Maruff P., O’Regan M.K., Papas A., Stephens T.C.B. (2019). The Vitamins in Psychosis Study: A Randomized, Double-Blind, Placebo-Controlled Trial of the Effects of Vitamins B12, B6, and Folic Acid on Symptoms and Neurocognition in First-Episode Psychosis. Biol. Psychiatry.

[39] Mullier E., Roine T., Griffa A., Xin L., Baumann P.S., Klauser P., Cleusix M., Jenni R., Alemàn-Gómez Y., Gruetter R. (2019). N-Acetyl-Cysteine Supplementation Improves Functional Connectivity within the Cingulate Cortex in Early Psychosis: A Pilot Study. Int. J. Neuropsychopharmacol..

[40] Szeszko P.R., McNamara R.K., Gallego J.A., Malhotra A.K., Govindarajulu U., Peters B.D., Robinson D.G. (2021). Longitudinal Investigation of the Relationship between Omega-3 Polyunsaturated Fatty Acids and Neuropsychological Functioning in Recent-Onset Psychosis: A Randomized Clinical Trial. Schizophr. Res..

[41] Pawełczyk T., Grancow-Grabka M., Żurner N., Pawełczyk A. (2021). Omega-3 Fatty Acids Reduce Cardiometabolic Risk in First-Episode Schizophrenia Patients Treated with Antipsychotics: Findings from the OFFER Randomized Controlled Study. Schizophr. Res..

[42] Gaughran F., Stringer D., Wojewodka G., Landau S., Smith S., Gardner-Sood P., Taylor D., Jordan H., Whiskey E., Krivoy A. (2021). Effect of Vitamin D Supplementation on Outcomes in People with Early Psychosis: The DFEND Randomized Clinical Trial. JAMA Netw. Open.

[43] Lyall A.E., Nägele F.L., Pasternak O., Gallego J.A., Malhotra A.K., McNamara R.K., Kubicki M., Peters B.D., Robinson D.G., Szeszko P.R. (2021). A 16-Week Randomized Placebo-Controlled Trial Investigating the Effects of Omega-3 Polyunsaturated Fatty Acid Treatment on White Matter Microstructure in Recent-Onset Psychosis Patients Concurrently Treated with Risperidone. Psychiatry Res. Neuroimaging.

[44] Huang J., Kang D., Zhang F., Yang Y., Liu C., Xiao J., Long Y., Lang B., Peng X., Wang W. (2022). Probiotics Plus Dietary Fiber Supplements Attenuate Olanzapine-Induced Weight Gain in Drug-Naïve First-Episode Schizophrenia Patients: Two Randomized Clinical Trials. Schizophr. Bull..

[45] Jamilian H., Solhi H., Jamilian M. (2014). Randomized, Placebo-Controlled Clinical Trial of Omega-3 as Supplemental Treatment in Schizophrenia. Glob. J. Health Sci..

[46] Sanders L.L.O., de Souza Menezes C.E., Chaves Filho A.J.M., de Almeida Viana G., Fechine F.V., Rodrigues de Queiroz M.G., Gonçalvez da Cruz Fonseca S., Mendes Vasconcelos S.M., Amaral de Moraes M.E., Gama C.S. (2017). α-Lipoic Acid as Adjunctive Treatment for Schizophrenia: An Open-Label Trial. J. Clin. Psychopharmacol..

[47] Qiao Y., Mei Y., Han H., Liu F., Yang X.M., Shao Y., Xie B., Long B. (2018). Effects of Omega-3 in the Treatment of Violent Schizophrenia Patients. Schizophr. Res..

[48] Robinson D.G., Gallego J.A., John M., Hanna L.A., Zhang J.P., Birnbaum M.L., Greenberg J., Naraine M., Peters B.D., McNamara R.K. (2019). A Potential Role for Adjunctive Omega-3 Polyunsaturated Fatty Acids for Depression and Anxiety Symptoms in Recent Onset Psychosis: Results from a 16 week Randomized Placebo-Controlled Trial for Participants Concurrently Treated with Risperidone. Schizophr. Res..

[49] Ghaderi A., Banafshe H.R., Mirhosseini N., Moradi M., Karimi M.-A., Mehrzad F., Bahmani F., Asemi Z. (2019). Clinical and Metabolic Response to Vitamin D plus Probiotic in Schizophrenia Patients. BMC Psychiatry.

[50] Xu F., Fan W., Wang W., Tang W., Yang F., Zhang Y., Cai J., Song L., Zhang C. (2019). Effects of Omega-3 Fatty Acids on Metabolic Syndrome in Patients with Schizophrenia: A 12-Week Randomized Placebo-Controlled Trial. Psychopharmacology.

[51] Tang W., Wang Y., Xu F., Fan W., Zhang Y., Fan K., Wang W., Zhang Y., Zhang C. (2020). Omega-3 Fatty Acids Ameliorate Cognitive Dysfunction in Schizophrenia Patients with Metabolic Syndrome. Brain Behav. Immun..

[52] Maguire Á., Mooney C., Flynn G., Ferguson Y., O’Keane V., O’Rourke D., McMonagle T., Heaton R., Phillips S., Hargreaves I. (2021). No Effect of Coenzyme Q10 on Cognitive Function, Psychological Symptoms, and Health-Related Outcomes in Schizophrenia and Schizoaffective Disorder: Results of a Randomized, Placebo-Controlled Trial. J. Clin. Psychopharmacol..

[53] Jamilian H., Ghaderi A. (2021). The Effects of Probiotic and Selenium Co-Supplementation on Clinical and Metabolic Scales in Chronic Schizophrenia: A Randomized, Double-Blind, Placebo-Controlled Trial. Biol. Trace Elem. Res..

[54] Mishra A., Reeta K.H., Sarangi S.C., Maiti R., Sood M. (2022). Effect of Add-on Alpha Lipoic Acid on Psychopathology in Patients with Treatment-Resistant Schizophrenia: A Pilot Randomized Double-Blind Placebo-Controlled Trial. Psychopharmacology.

[55] Sevillano-Jiménez A., Romero-Saldaña M., García-Mellado J.A., Carrascal-Laso L., García-Rodríguez M., Molina-Luque R., Molina-Recio G. (2022). Impact of High Prebiotic and Probiotic Dietary Education in the SARS-CoV-2 Era: Improved Cardio-Metabolic Profile in Schizophrenia Spectrum Disorders. BMC Psychiatry.

[56] De Lima D.N., Costa Filho C.W.L., Frota I.J., de Oliveira A.L.B., Menezes C.E. (2023). de S.; Chaves Filho, A.J.M.; Viana, G. de A.; Campos, E. de M.; Collares, M.; de Queiroz, M.G.R.; et al. α-Lipoic Acid as Adjunctive Treatment for Schizophrenia: A Randomized Double-Blind Study. J. Clin. Psychopharmacol..

[57] Kalejahi P., Kheirouri S., Noorazar S.G. (2023). A Randomized Controlled Trial of Vitamin D Supplementation in Iranian Patients with Schizophrenia: Effects on Serum Levels of Glycogen Synthase Kinase-3β and Symptom Severity. Int. J. Psychiatry Med..

[58] Bent S., Hendren R.L., Zandi T., Law K., Choi J.-E., Widjaja F., Kalb L., Nestle J., Law P. (2014). Internet-Based, Randomized, Controlled Trial of Omega-3 Fatty Acids for Hyperactivity in Autism. J. Am. Acad. Child Adolesc. Psychiatry.

[59] Voigt R.G., Mellon M.W., Katusic S.K., Weaver A.L., Matern D., Mellon B., Jensen C.L., Barbaresi W.J. (2014). Dietary Docosahexaenoic Acid Supplementation in Children with Autism. J. Pediatr. Gastroenterol. Nutr..

[60] Mankad D., Dupuis A., Smile S., Roberts W., Brian J., Lui T., Genore L., Zaghloul D., Iaboni A., Marcon P.M.A. (2015). A Randomized, Placebo Controlled Trial of Omega-3 Fatty Acids in the Treatment of Young Children with Autism. Mol. Autism.

[61] Ooi Y.P., Weng S.-J., Jang L.Y., Low L., Seah J., Teo S., Ang R.P., Lim C.G., Liew A., Fung D.S. (2015). Omega-3 Fatty Acids in the Management of Autism Spectrum Disorders: Findings from an Open-Label Pilot Study in Singapore. Eur. J. Clin. Nutr..

[62] Tomova A., Husarova V., Lakatosova S., Bakos J., Vlkova B., Babinska K., Ostatnikova D. (2015). Gastrointestinal Microbiota in Children with Autism in Slovakia. Physiol. Behav..

[63] Grossi E., Melli S., Dunca D., Terruzzi V. (2016). Unexpected Improvement in Core Autism Spectrum Disorder Symptoms after Long-Term Treatment with Probiotics. SAGE Open Med. Case Rep..

[64] Sheppard K.W., Boone K.M., Gracious B., Klebanoff M.A., Rogers L.K., Rausch J., Bartlett C., Coury D.L., Keim S.A. (2017). Effect of Omega-3 and -6 Supplementation on Language in Preterm Toddlers Exhibiting Autism Spectrum Disorder Symptoms. J. Autism Dev. Disord..

[65] Kang D.-W., Adams J.B., Gregory A.C., Borody T., Chittick L., Fasano A., Khoruts A., Geis E., Maldonado J., McDonough-Means S. (2017). Microbiota Transfer Therapy Alters Gut Ecosystem and Improves Gastrointestinal and Autism Symptoms: An Open-Label Study. Microbiome.

[66] Parellada M., Llorente C., Calvo R., Gutierrez S., Lázaro L., Graell M., Guisasola M., Dorado M.L., Boada L., Romo J. (2017). Randomized Trial of Omega-3 for Autism Spectrum Disorders: Effect on Cell Membrane Composition and Behavior. Eur. Neuropsychopharmacol..

[67] Keim S.A., Gracious B., Boone K.M., Klebanoff M.A., Rogers L.K., Rausch J., Coury D.L., Sheppard K.W., Husk J., Rhoda D.A. (2018). ω-3 and ω-6 Fatty Acid Supplementation May Reduce Autism Symptoms Based on Parent Report in Preterm Toddlers. J. Nutr..

[68] Mazahery H., Conlon C.A., Beck K.L., Mugridge O., Kruger M.C., Stonehouse W., Camargo C.A., Meyer B.J., Jones B., von Hurst P.R. (2019). A Randomised Controlled Trial of Vitamin D and Omega-3 Long Chain Polyunsaturated Fatty Acids in the Treatment of Irritability and Hyperactivity among Children with Autism Spectrum Disorder. J. Steroid Biochem. Mol. Biol..

[69] Liu Y.-W., Liong M.T., Chung Y.-C.E., Huang H.-Y., Peng W.-S., Cheng Y.-F., Lin Y.-S., Wu Y.-Y., Tsai Y.-C. (2019). Effects of Lactobacillus Plantarum PS128 on Children with Autism Spectrum Disorder in Taiwan: A Randomized, Double-Blind, Placebo-Controlled Trial. Nutrients.

[70] Wang Y., Li N., Yang J.-J., Zhao D.-M., Chen B., Zhang G.-Q., Chen S., Cao R.-F., Yu H., Zhao C.-Y. (2020). Probiotics and Fructo-Oligosaccharide Intervention Modulate the Microbiota-Gut Brain Axis to Improve Autism Spectrum Reducing Also the Hyper-Serotonergic State and the Dopamine Metabolism Disorder. Pharmacol. Res..

[71] Javadfar Z., Abdollahzad H., Moludi J., Rezaeian S., Amirian H., Foroughi A.A., Nachvak S.M., Goharmehr N., Mostafai R. (2020). Effects of Vitamin D Supplementation on Core Symptoms, Serum Serotonin, and Interleukin-6 in Children with Autism Spectrum Disorders: A Randomized Clinical Trial. Nutrition.

[72] Boone K.M., Parrott A., Rausch J., Yeates K.O., Klebanoff M.A., Norris Turner A., Keim S.A. (2020). Fatty Acid Supplementation and Socioemotional Outcomes: Secondary Analysis of a Randomized Trial. Pediatrics.

[73] Renard E., Leheup B., Guéant-Rodriguez R.-M., Oussalah A., Quadros E.V., Guéant J.-L. (2020). Folinic Acid Improves the Score of Autism in the EFFET Placebo-Controlled Randomized Trial. Biochimie.

[74] Kong X.-J., Liu J., Liu K., Koh M., Sherman H., Liu S., Tian R., Sukijthamapan P., Wang J., Fong M. (2021). Probiotic and Oxytocin Combination Therapy in Patients with Autism Spectrum Disorder: A Randomized, Double-Blinded, Placebo-Controlled Pilot Trial. Nutrients.

[75] Doaei S., Bourbour F., Teymoori Z., Jafari F., Kalantari N., Abbas Torki S., Ashoori N., Nemat Gorgani S., Gholamalizadeh M. (2021). The Effect of Omega-3 Fatty Acids Supplementation on Social and Behavioral Disorders of Children with Autism: A Randomized Clinical Trial. Pediatr. Endocrinol. Diabetes Metab..

[76] Batebi N., Moghaddam H.S., Hasanzadeh A., Fakour Y., Mohammadi M.R., Akhondzadeh S. (2021). Folinic Acid as Adjunctive Therapy in Treatment of Inappropriate Speech in Children with Autism: A Double-Blind and Placebo-Controlled Randomized Trial. Child Psychiatry Hum. Dev..

[77] Boone K.M., Klebanoff M.A., Rogers L.K., Rausch J., Coury D.L., Keim S.A. (2022). Effects of Omega-3-6-9 Fatty Acid Supplementation on Behavior and Sleep in Preterm Toddlers with Autism Symptomatology: Secondary Analysis of a Randomized Clinical Trial. Early Hum. Dev..

[78] Keim S.A., Jude A., Smith K., Khan A.Q., Coury D.L., Rausch J., Udaipuria S., Norris M., Bartram L.R., Narayanan A.R. (2022). Randomized Controlled Trial of Omega-3 and -6 Fatty Acid Supplementation to Reduce Inflammatory Markers in Children with Autism Spectrum Disorder. J. Autism Dev. Disord..

[79] Schmitt L.M., Smith E.G., Pedapati E.V., Horn P.S., Will M., Lamy M., Barber L., Trebley J., Meyer K., Heiman M. (2023). Results of a Phase Ib Study of SB-121, an Investigational Probiotic Formulation, a Randomized Controlled Trial in Participants with Autism Spectrum Disorder. Sci. Rep..

[80] Ginty A.T., Conklin S.M. (2015). Short-Term Supplementation of Acute Long-Chain Omega-3 Polyunsaturated Fatty Acids May Alter Depression Status and Decrease Symptomology among Young Adults with Depression: A Preliminary Randomized and Placebo Controlled Trial. Psychiatry Res..

[81] Mischoulon D., Nierenberg A.A., Schettler P.J., Kinkead B.L., Fehling K., Martinson M.A., Hyman Rapaport M. (2015). A Double-Blind, Randomized Controlled Clinical Trial Comparing Eicosapentaenoic Acid versus Docosahexaenoic Acid for Depression. J. Clin. Psychiatry.

[82] Park Y., Park Y.-S., Kim S.H., Oh D.H., Park Y.-C. (2015). Supplementation of N-3 Polyunsaturated Fatty Acids for Major Depressive Disorder: A Randomized, Double-Blind, 12-Week, Placebo-Controlled Trial in Korea. Ann. Nutr. Metab..

[83] Rapaport M.H., Nierenberg A.A., Schettler P.J., Kinkead B., Cardoos A., Walker R., Mischoulon D. (2016). Inflammation as a Predictive Biomarker for Response to Omega-3 Fatty Acids in Major Depressive Disorder: A Proof-of-Concept Study. Mol. Psychiatry.

[84] Young A.S., Arnold L.E., Wolfson H.L., Fristad M.A. (2017). Psychoeducational Psychotherapy and Omega-3 Supplementation Improve Co-Occurring Behavioral Problems in Youth with Depression: Results from a Pilot RCT. J. Abnorm. Child Psychol..

[85] Gabbay V., Freed R.D., Alonso C.M., Senger S., Stadterman J., Davison B.A., Klein R.G. (2018). A Double-Blind Placebo-Controlled Trial of Omega-3 Fatty Acids as a Monotherapy for Adolescent Depression. J. Clin. Psychiatry.

[86] Jahangard L., Sadeghi A., Ahmadpanah M., Holsboer-Trachsler E., Sadeghi Bahmani D., Haghighi M., Brand S. (2018). Influence of Adjuvant Omega-3-Polyunsaturated Fatty Acids on Depression, Sleep, and Emotion Regulation among Outpatients with Major Depressive Disorders—Results from a Double-Blind, Randomized and Placebo-Controlled Clinical Trial. J. Psychiatr. Res..

[87] Hansen J.P., Pareek M., Hvolby A., Schmedes A., Toft T., Dahl E., Nielsen C.T. (2019). Vitamin D3 Supplementation and Treatment Outcomes in Patients with Depression (D3-Vit-Dep). BMC Res. Notes.

[88] Tayama J., Ogawa S., Nakaya N., Sone T., Hamaguchi T., Takeoka A., Hamazaki K., Okamura H., Yajima J., Kobayashi M. (2019). Omega-3 Polyunsaturated Fatty Acids and Psychological Intervention for Workers with Mild to Moderate Depression: A Double-Blind Randomized Controlled Trial. J. Affect. Disord..

[89] Chahwan B., Kwan S., Isik A., van Hemert S., Burke C., Roberts L. (2019). Gut Feelings: A Randomised, Triple-Blind, Placebo-Controlled Trial of Probiotics for Depressive Symptoms. J. Affect. Disord..

[90] Parletta N., Zarnowiecki D., Cho J., Wilson A., Bogomolova S., Villani A., Itsiopoulos C., Niyonsenga T., Blunden S., Meyer B. (2019). A Mediterranean-Style Dietary Intervention Supplemented with Fish Oil Improves Diet Quality and Mental Health in People with Depression: A Randomized Controlled Trial (HELFIMED). Nutr. Neurosci..

[91] Karakula-Juchnowicz H., Rog J., Juchnowicz D., Łoniewski I., Skonieczna-Żydecka K., Krukow P., Futyma-Jedrzejewska M., Kaczmarczyk M. (2019). The Study Evaluating the Effect of Probiotic Supplementation on the Mental Status, Inflammation, and Intestinal Barrier in Major Depressive Disorder Patients Using Gluten-Free or Gluten-Containing Diet (SANGUT Study): A 12-Week, Randomized, Double-Blind, and Placebo-Controlled Clinical Study Protocol. Nutr. J..

[92] Kazemi A., Noorbala A.A., Azam K., Eskandari M.H., Djafarian K. (2019). Effect of Probiotic and Prebiotic vs Placebo on Psychological Outcomes in Patients with Major Depressive Disorder: A Randomized Clinical Trial. Clin. Nutr..

[93] de Koning E.J., Lips P., Penninx B.W.J.H., Elders P.J.M., Heijboer A.C., den Heijer M., Bet P.M., van Marwijk H.W.J., van Schoor N.M. (2019). Vitamin D Supplementation for the Prevention of Depression and Poor Physical Function in Older Persons: The D-Vitaal Study, a Randomized Clinical Trial. Am. J. Clin. Nutr..

[94] Alavi N.M., Khademalhoseini S., Vakili Z., Assarian F. (2019). Effect of Vitamin D Supplementation on Depression in Elderly Patients: A Randomized Clinical Trial. Clin. Nutr..

[95] Saccarello A., Montarsolo P., Massardo I., Picciotto R., Pedemonte A., Castagnaro R., Brasesco P.C., Guida V., Picco P., Fioravanti P. (2020). Oral Administration of S-Adenosylmethionine (SAMe) and Lactobacillus Plantarum HEAL9 Improves the Mild-To-Moderate Symptoms of Depression: A Randomized, Double-Blind, Placebo-Controlled Study. Prim. Care Companion CNS Disord..

[96] Trebatická J., Hradečná Z., Surovcová A., Katrenčíková B., Gushina I., Waczulíková I., Sušienková K., Garaiova I., Šuba J., Ďuračková Z. (2020). Omega-3 Fatty-Acids Modulate Symptoms of Depressive Disorder, Serum Levels of Omega-3 Fatty Acids and Omega-6/Omega-3 Ratio in Children. A Randomized, Double-Blind and Controlled Trial. Psychiatry Res..

[97] Reininghaus E.Z., Platzer M., Kohlhammer-Dohr A., Hamm C., Mörkl S., Bengesser S.A., Fellendorf F.T., Lahousen-Luxenberger T., Leitner-Afschar B., Schöggl H. (2020). PROVIT: Supplementary Probiotic Treatment and Vitamin B7 in Depression-A Randomized Controlled Trial. Nutrients.

[98] Reiter A., Bengesser S.A., Hauschild A.-C., Birkl-Töglhofer A.-M., Fellendorf F.T., Platzer M., Färber T., Seidl M., Mendel L.-M., Unterweger R. (2020). Interleukin-6 Gene Expression Changes after a 4-Week Intake of a Multispecies Probiotic in Major Depressive Disorder-Preliminary Results of the PROVIT Study. Nutrients.

[99] Kaviani M., Nikooyeh B., Zand H., Yaghmaei P., Neyestani T.R. (2020). Effects of Vitamin D Supplementation on Depression and Some Involved Neurotransmitters. J. Affect. Disord..

[100] Zhu C., Zhang Y., Wang T., Lin Y., Yu J., Xia Q., Zhu P., Zhu D.-M. (2020). Vitamin D Supplementation Improves Anxiety but Not Depression Symptoms in Patients with Vitamin D Deficiency. Brain Behav..

[101] Libuda L., Timmesfeld N., Antel J., Hirtz R., Bauer J., Führer D., Zwanziger D., Öztürk D., Langenbach G., Hahn D. (2020). Effect of Vitamin D Deficiency on Depressive Symptoms in Child and Adolescent Psychiatric Patients: Results of a Randomized Controlled Trial. Eur. J. Nutr..

[102] Ho Y.-T., Tsai Y.-C., Kuo T.B.J., Yang C.C.H. (2021). Effects of Lactobacillus Plantarum PS128 on Depressive Symptoms and Sleep Quality in Self-Reported Insomniacs: A Randomized, Double-Blind, Placebo-Controlled Pilot Trial. Nutrients.

[103] Lee H.J., Hong J.K., Kim J.-K., Kim D.-H., Jang S.W., Han S.-W., Yoon I.-Y. (2021). Effects of Probiotic NVP-1704 on Mental Health and Sleep in Healthy Adults: An 8-Week Randomized, Double-Blind, Placebo-Controlled Trial. Nutrients.

[104] Mischoulon D., Dunlop B.W., Kinkead B., Schettler P.J., Lamon-Fava S., Rakofsky J.J., Nierenberg A.A., Clain A.J., Mletzko Crowe T., Wong A. (2022). Omega-3 Fatty Acids for Major Depressive Disorder with High Inflammation: A Randomized Dose-Finding Clinical Trial. J. Clin. Psychiatry.

[105] Kaviani M., Nikooyeh B., Etesam F., Behnagh S.J., Kangarani H.M., Arefi M., Yaghmaei P., Neyestani T.R. (2022). Effects of Vitamin D Supplementation on Depression and Some Selected Pro-Inflammatory Biomarkers: A Double-Blind Randomized Clinical Trial. BMC Psychiatry.

[106] Schaub A.-C., Schneider E., Vazquez-Castellanos J.F., Schweinfurth N., Kettelhack C., Doll J.P.K., Yamanbaeva G., Mählmann L., Brand S., Beglinger C. (2022). Clinical, Gut Microbial and Neural Effects of a Probiotic Add-on Therapy in Depressed Patients: A Randomized Controlled Trial. Transl. Psychiatry.

[107] Ullah H., Di Minno A., Esposito C., El-Seedi H.R., Khalifa S.A.M., Baldi A., Greco A., Santonastaso S., Cioffi V., Sperandeo R. (2022). Efficacy of a Food Supplement Based on S-Adenosyl Methionine and Probiotic Strains in Subjects with Subthreshold Depression and Mild-to-Moderate Depression: A Monocentric, Randomized, Cross-over, Double-Blind, Placebo-Controlled Clinical Trial. Biomed. Pharmacother..

[108] Schneider E., Doll J.P.K., Schweinfurth N., Kettelhack C., Schaub A.-C., Yamanbaeva G., Varghese N., Mählmann L., Brand S., Eckert A. (2023). Effect of Short-Term, High-Dose Probiotic Supplementation on Cognition, Related Brain Functions and BDNF in Patients with Depression: A Secondary Analysis of a Randomized Controlled Trial. J. Psychiatry Neurosci..

[109] Nikolova V.L., Cleare A.J., Young A.H., Stone J.M. (2023). Acceptability, Tolerability, and Estimates of Putative Treatment Effects of Probiotics as Adjunctive Treatment in Patients with Depression: A Randomized Clinical Trial. JAMA Psychiatry.

[110] Zhu R., Fang Y., Li H., Liu Y., Wei J., Zhang S., Wang L., Fan R., Wang L., Li S. (2023). Psychobiotic Lactobacillus Plantarum JYLP-326 Relieves Anxiety, Depression, and Insomnia Symptoms in Test Anxious College via Modulating the Gut Microbiota and Its Metabolism. Front. Immunol..

[111] Sharpley A.L., Hockney R., McPeake L., Geddes J.R., Cowen P.J. (2014). Folic Acid Supplementation for Prevention of Mood Disorders in Young People at Familial Risk: A Randomised, Double Blind, Placebo Controlled Trial. J. Affect. Disord..

[112] Fristad M.A., Young A.S., Vesco A.T., Nader E.S., Healy K.Z., Gardner W., Wolfson H.L., Arnold L.E. (2015). A Randomized Controlled Trial of Individual Family Psychoeducational Psychotherapy and Omega-3 Fatty Acids in Youth with Subsyndromal Bipolar Disorder. J. Child Adolesc. Psychopharmacol..

[113] Wozniak J., Faraone S., Chan J., Tarko L., Hernandez M., Davis J., Woodworth Y., Biederman J. (2016). Correction: A Randomized Clinical Trial of High Eicosapentaenoic Acid Omega-3 Fatty Acids and Inositol as Monotherapy and in Combination in the Treatment of Pediatric Bipolar Spectrum Disorders: A Pilot Study. J. Clin. Psychiatry.

[114] Marsh W.K., Penny J.L., Rothschild A.J. (2017). Vitamin D Supplementation in Bipolar Depression: A Double Blind Placebo Controlled Trial. J. Psychiatr. Res..

[115] Nierenberg A.A., Montana R., Kinrys G., Deckersbach T., Dufour S., Baek J.H. (2017). L-Methylfolate For Bipolar I Depressive Episodes: An Open Trial Proof-of-Concept Registry. J. Affect. Disord..

[116] Dickerson F., Adamos M., Katsafanas E., Khushalani S., Origoni A., Savage C., Schweinfurth L., Stallings C., Sweeney K., Goga J. (2018). Adjunctive Probiotic Microorganisms to Prevent Rehospitalization in Patients with Acute Mania: A Randomized Controlled Trial. Bipolar. Disord..

[117] Mehrpooya M., Yasrebifar F., Haghighi M., Mohammadi Y., Jahangard L. (2018). Evaluating the Effect of Coenzyme Q10 Augmentation on Treatment of Bipolar Depression: A Double-Blind Controlled Clinical Trial. J. Clin. Psychopharmacol..

[118] Vesco A.T., Young A.S., Arnold L.E., Fristad M.A. (2018). Omega-3 Supplementation Associated with Improved Parent-Rated Executive Function in Youth with Mood Disorders: Secondary Analyses of the Omega-3 and Therapy (OATS) Trials. J. Child Psychol. Psychiatry Allied Discip..

[119] Toniolo R.A., Silva M., Fernandes F. (2018). de B.F.; Amaral, J.A. de M.S.; Dias, R. da S.; Lafer, B. A Randomized, Double-Blind, Placebo-Controlled, Proof-of-Concept Trial of Creatine Monohydrate as Adjunctive Treatment for Bipolar Depression. J. Neural. Transm..

[120] McNamara R.K., Strawn J.R., Tallman M.J., Welge J.A., Patino L.R., Blom T.J., DelBello M.P. (2020). Effects of Fish Oil Monotherapy on Depression and Prefrontal Neurochemistry in Adolescents at High Risk for Bipolar I Disorder: A 12-Week Placebo-Controlled Proton Magnetic Resonance Spectroscopy Trial. J. Child Adolesc. Psychopharmacol..

[121] Ashton M.M., Mohebbi M., Turner A., Marx W., Berk M., Malhi G.S., Ng C.H., Cotton S.M., Dodd S., Sarris J. (2020). Physical Activity as a Predictor of Clinical Trial Outcomes in Bipolar Depression: A Subanalysis of a Mitochondrial-Enhancing Nutraceutical Randomized Controlled Trial. Can. J. Psychiatry.

[122] McPhilemy G., Byrne F., Waldron M., Hibbeln J.R., Davis J., McDonald C., Hallahan B. (2021). A 52-Week Prophylactic Randomised Control Trial of Omega-3 Polyunsaturated Fatty Acids in Bipolar Disorder. Bipolar. Disord..

[123] Badrfam R., Mostafavi S.-A., Khaleghi A., Akhondzadeh S., Zandifar A., Farid M., Mohammadian Khonsari N., Mohammadi M.R. (2021). The Efficacy of Vitamin B6 as an Adjunctive Therapy to Lithium in Improving the Symptoms of Acute Mania in Patients with Bipolar Disorder, Type 1; a Double-Blind, Randomized, Placebo-Controlled, Clinical Trial. Brain Behav..

[124] Fristad M.A., Roley-Roberts M.E., Black S.R., Arnold L.E. (2021). Moody Kids Years Later: Long-Term Outcomes of Youth from the Omega-3 and Therapy (OATS) Studies. J. Affect. Disord..

[125] Sabouri S., Esmailzadeh M., Sadeghinejad A., Eslami Shahrbabaki M., Asadikaram G., Nikvarz N. (2022). The Effect of Adjunctive Probiotics on Markers of Inflammation and Oxidative Stress in Bipolar Disorder: A Double-Blind, Randomized, Controlled Trial. J. Psychiatr. Pract..

[126] McNamara R.K., Li W., Lei D., Tallman M.J., Welge J.A., Strawn J.R., Patino L.R., DelBello M.P. (2022). Fish Oil Supplementation Alters Emotion-Generated Corticolimbic Functional Connectivity in Depressed Adolescents at High-Risk for Bipolar I Disorder: A 12-Week Placebo-Controlled fMRI Trial. Bipolar Disord..

[127] Saunders E.F.H., Mukherjee D., Myers T., Wasserman E., Hameed A., Bassappa Krishnamurthy V., MacIntosh B., Domenichiello A., Ramsden C.E., Wang M. (2022). Adjunctive Dietary Intervention for Bipolar Disorder: A Randomized, Controlled, Parallel-Group, Modified Double-Blinded Trial of a High n-3 plus Low n-6 Diet. Bipolar. Disord..

[128] Wozniak J., Farrell A., DiSalvo M., Ceranoglu A., Uchida M., Vaudreuil C., Joshi G., Faraone S.V., Cook E., Biederman J. (2022). A Randomized, Double-Blind, Controlled Clinical Trial of Omega-3 Fatty Acids and Inositol as Monotherapies and in Combination for the Treatment of Pediatric Bipolar Spectrum Disorder in Children Age 5-12. Psychopharmacol. Bull..

[129] Eslahi H., Shakiba M., Saravani M., Payandeh A., Shahraki M. (2023). The Effects of Omega-3 Fatty Acids on the Serum Concentrations of pro Inflammatory Cytokines Anddepression Status in Patients with Bipolar Disorder: A Randomized Double-Blind Controlled Clinical Trial. J. Res. Med. Sci..

[130] Zailani H., Wu S.-K., Yang K.-J., Malau I.A., Liao H.-F., Chung Y.-L., Chang J.P.-C., Chiu W.-C., Su K.-P. (2024). Omega-3 Polyunsaturated Fatty Acids in the Prevention of Relapse in Patients with Stable Bipolar Disorder: A 6-Month Pilot Randomized Controlled Trial. Psychiatry Res..

[131] Zandifar A., Mousavi S., Schmidt N.B., Badrfam R., Seif E., Qorbani M., Mehrabani Natanzi M. (2024). Efficacy of Vitamins B1 and B6 as an Adjunctive Therapy to Lithium in Bipolar-I Disorder: A Double-Blind, Randomized, Placebo-Controlled, Clinical Trial. J. Affect. Disord..

[132] Bellino S., Bozzatello P., Rocca G., Bogetto F. (2014). Efficacy of Omega-3 Fatty Acids in the Treatment of Borderline Personality Disorder: A Study of the Association with Valproic Acid. J. Psychopharmacol..

[133] Bozzatello P., Rocca P., Bellino S. (2018). Combination of Omega-3 Fatty Acids and Valproic Acid in Treatment of Borderline Personality Disorder: A Follow-Up Study. Clin. Drug Investig..

[134] Raine A., Fung A.L.C., Gao Y., Lee T.M.C. (2021). Omega-3 Supplementation, Child Antisocial Behavior, and Psychopathic Personality: A Randomized, Double-Blind, Placebo-Controlled, Stratified, Parallel Group Trial. Eur. Child Adolesc. Psychiatry.

[135] Andreasen N.C., Flaum M. (1991). Schizophrenia: The Characteristic Symptoms. Schizophr. Bull..

[136] Campana M., Falkai P., Siskind D., Hasan A., Wagner E. (2021). Characteristics and Definitions of Ultra-Treatment-Resistant Schizophrenia—A Systematic Review and Meta-Analysis. Schizophr. Res..

[137] Carruthers S.P., Van Rheenen T.E., Karantonis J.A., Rossell S.L. (2022). Characterising Demographic, Clinical and Functional Features of Cognitive Subgroups in Schizophrenia Spectrum Disorders: A Systematic Review. Neuropsychol. Rev..

[138] Wolkin A., Segarnick D., Sierkierski J., Manku M., Horrobin D., Rotrosen J. (1987). Essential Fatty Acid Supplementation during Early Alcohol Abstinence. Alcohol. Clin. Exp. Res..

[139] Fenton W.S., Hibbeln J., Knable M. (2000). Essential Fatty Acids, Lipid Membrane Abnormalities, and the Diagnosis and Treatment of Schizophrenia. Biol. Psychiatry.

[140] Berger M., Nelson B., Markulev C., Yuen H.P., Schäfer M.R., Mossaheb N., Schlögelhofer M., Smesny S., Hickie I.B., Berger G.E. (2019). Relationship between Polyunsaturated Fatty Acids and Psychopathology in the NEURAPRO Clinical Trial. Front. Psychiatry.

[141] Glen A.I., Glen E.M., Horrobin D.F., Vaddadi K.S., Spellman M., Morse-Fisher N., Ellis K., Skinner F.S. (1994). A Red Cell Membrane Abnormality in a Subgroup of Schizophrenic Patients: Evidence for Two Diseases. Schizophr. Res..

[142] Assies J., Lieverse R., Vreken P., Wanders R.J.A., Dingemans P.M.J.A., Linszen D.H. (2001). Significantly Reduced Docosahexaenoic and Docosapentaenoic Acid Concentrations in Erythrocyte Membranes from Schizophrenic Patients Compared with a Carefully Matched Control Group. Biol. Psychiatry.

[143] Reddy R.D., Keshavan M.S., Yao J.K. (2004). Reduced Red Blood Cell Membrane Essential Polyunsaturated Fatty Acids in First Episode Schizophrenia at Neuroleptic-Naive Baseline. Schizophr. Bull..

[144] Schlögelhofer M., Amminger G.P., Schaefer M.R., Fusar-Poli P., Smesny S., McGorry P., Berger G., Mossaheb N. (2014). Polyunsaturated Fatty Acids in Emerging Psychosis: A Safer Alternative?. Early Interv. Psychiatry.

[145] Cadenhead K.S., Minichino A., Kelsven S., Addington J., Bearden C., Cannon T.D., Cornblatt B.A., Mathalon D., McGlashan T.H., Perkins D.O. (2019). Metabolic Abnormalities and Low Dietary Omega-3 Are Associated with Symptom Severity and Worse Functioning Prior to the Onset of Psychosis: Findings from the North American Prodrome Longitudinal Studies Consortium. Schizophr. Res..

[146] Sethom M.M., Fares S., Bouaziz N., Melki W., Jemaa R., Feki M., Hechmi Z., Kaabachi N. (2010). Polyunsaturated Fatty Acids Deficits Are Associated with Psychotic State and Negative Symptoms in Patients with Schizophrenia. Prostaglandins Leukot. Essent. Fat. Acids.

[147] Sumiyoshi T., Higuchi Y., Matsui M., Itoh H., Uehara T., Itoh T., Arai H., Takamiya C., Suzuki M., Kurachi M. (2011). Membrane Fatty Acid Levels as a Predictor of Treatment Response in Chronic Schizophrenia. Psychiatry Res..

[148] Tessier C., Sweers K., Frajerman A., Bergaoui H., Ferreri F., Delva C., Lapidus N., Lamaziere A., Roiser J.P., De Hert M. (2016). Membrane Lipidomics in Schizophrenia Patients: A Correlational Study with Clinical and Cognitive Manifestations. Transl. Psychiatry.

[149] Yao J., Vankammen D. (1994). Red Blood Cell Membrane Dynamics in Schizophrenia I. Membrane Fluidity. Schizophr. Res..

[150] Marshall M., Rathbone J. (2006). Early Intervention for Psychosis. Cochrane Database of Systematic Reviews.

[151] Rapado-Castro M., McGorry P.D., Yung A., Calvo A., Nelson B. (2015). Sources of Clinical Distress in Young People at Ultra High Risk of Psychosis. Schizophr. Res..

[152] Power L., Polari A.R., Yung A.R., Mcgorry P.D., Nelson B. (2016). Distress in Relation to Attenuated Psychotic Symptoms in the Ultra-High-Risk Population Is Not Associated with Increased Risk of Psychotic Disorder. Early Interv. Psychiatry.

[153] Ziermans T.B., Schothorst P.F., Sprong M., van Engeland H. (2011). Transition and Remission in Adolescents at Ultra-High Risk for Psychosis. Schizophr. Res..

[154] McGlashan T.H., Zipursky R.B., Perkins D., Addington J., Miller T., Woods S.W., Hawkins K.A., Hoffman R.E., Preda A., Epstein I. (2006). Randomized, Double-Blind Trial of Olanzapine versus Placebo in Patients Prodromally Symptomatic for Psychosis. Am. J. Psychiatry.

[155] Anderson G., Maes M. (2013). Schizophrenia: Linking Prenatal Infection to Cytokines, the Tryptophan Catabolite (TRYCAT) Pathway, NMDA Receptor Hypofunction, Neurodevelopment and Neuroprogression. Prog. Neuro-Psychopharmacol. Biol. Psychiatry.

[156] Baio J., Wiggins L., Christensen D.L., Maenner M.J., Daniels J., Warren Z., Kurzius-Spencer M., Zahorodny W., Robinson Rosenberg C., White T. (2018). Prevalence of Autism Spectrum Disorder Among Children Aged 8 Years—Autism and Developmental Disabilities Monitoring Network, 11 Sites, United States, 2014. MMWR Surveill. Summ..

[157] Majhi S., Kumar S., Singh L. (2023). A Review on Autism Spectrum Disorder: Pathogenesis, Biomarkers, Pharmacological and Non-Pharmacological Interventions. CNS Neurol. Disord. Drug Targets.

[158] De Angelis M., Francavilla R., Piccolo M., De Giacomo A., Gobbetti M. (2015). Autism Spectrum Disorders and Intestinal Microbiota. Gut Microbes.

[159] Saad K., Abdel-Rahman A.A., Elserogy Y.M., Al-Atram A.A., Cannell J.J., Bjørklund G., Abdel-Reheim M.K., Othman H.A.K., El-Houfey A.A., Abd El-Aziz N.H.R. (2016). Vitamin D Status in Autism Spectrum Disorders and the Efficacy of Vitamin D Supplementation in Autistic Children. Nutr. Neurosci..

[160] Hu T., Dong Y., He C., Zhao M., He Q. (2020). The Gut Microbiota and Oxidative Stress in Autism Spectrum Disorders (ASD). Oxid. Med. Cell. Longev..

[161] Karhu E., Zukerman R., Eshraghi R.S., Mittal J., Deth R.C., Castejon A.M., Trivedi M., Mittal R., Eshraghi A.A. (2020). Nutritional Interventions for Autism Spectrum Disorder. Nutr. Rev..

[162] Jiang Y., Dang W., Nie H., Kong X., Jiang Z., Guo J. (2023). Omega-3 Polyunsaturated Fatty Acids and/or Vitamin D in Autism Spectrum Disorders: A Systematic Review. Front. Psychiatry.

[163] Horvath A., Łukasik J., Szajewska H. (2017). ω-3 Fatty Acid Supplementation Does Not Affect Autism Spectrum Disorder in Children: A Systematic Review and Meta-Analysis. J. Nutr..

[164] Masi A., Glozier N., Dale R., Guastella A.J. (2017). The Immune System, Cytokines, and Biomarkers in Autism Spectrum Disorder. Neurosci. Bull..

[165] Ramaekers V.T., Quadros E.V., Sequeira J.M. (2013). Role of Folate Receptor Autoantibodies in Infantile Autism. Mol. Psychiatry.

[166] Lintas C. (2019). Linking Genetics to Epigenetics: The Role of Folate and Folate-Related Pathways in Neurodevelopmental Disorders. Clin. Genet..

[167] Johnson C.R., Handen B.L., Zimmer M., Sacco K. (2010). Polyunsaturated Fatty Acid Supplementation in Young Children with Autism. J. Dev. Phys. Disabil..

[168] Kuzniewicz M.W., Wi S., Qian Y., Walsh E.M., Armstrong M.A., Croen L.A. (2014). Prevalence and Neonatal Factors Associated with Autism Spectrum Disorders in Preterm Infants. J. Pediatr..

[169] Pritchard M.A., de Dassel T., Beller E., Bogossian F., Johnston L., Paynter J., Russo S., Scott J. (2016). Autism in Toddlers Born Very Preterm. Pediatrics.

[170] Verhaeghe L., Dereu M., Warreyn P., De Groote I., Vanhaesebrouck P., Roeyers H. (2016). Extremely Preterm Born Children at Very High Risk for Developing Autism Spectrum Disorder. Child Psychiatry Hum. Dev..

[171] Girone N., Benatti B., Molteni L., Cassina N., Giacovelli L., Arici C., Dell’Osso B. (2023). Partial Response to Antidepressant Treatment: The Role of Nutraceutical Compounds. Clin. Neuropsychiatry.

[172] Peet M., Murphy B., Shay J., Horrobin D. (1998). Depletion of Omega-3 Fatty Acid Levels in Red Blood Cell Membranes of Depressive Patients. Biol. Psychiatry.

[173] Lin P.Y., Huang S.Y., Su K.P. (2010). A Meta-Analytic Review of Polyunsaturated Fatty Acid Compositions in Patients with Depression. Biol. Psychiatry.

[174] Hoffmire C.A., Block R.C., Thevenet-Morrison K., van Wijngaarden E. (2012). Associations between Omega-3 Poly-Unsaturated Fatty Acids from Fish Consumption and Severity of Depressive Symptoms: An Analysis of the 2005-2008 National Health and Nutrition Examination Survey. Prostaglandins Leukot. Essent. Fat. Acids.

[175] Beydoun M.A., Fanelli Kuczmarski M.T., Beydoun H.A., Hibbeln J.R., Evans M.K., Zonderman A.B. (2013). ω-3 Fatty Acid Intakes Are Inversely Related to Elevated Depressive Symptoms among United States Women. J. Nutr..

[176] Lotrich F.E. (2015). Inflammatory Cytokine-Associated Depression. Brain Res..

[177] Song C., Shieh C.-H., Wu Y.-S., Kalueff A., Gaikwad S., Su K.-P. (2016). The Role of Omega-3 Polyunsaturated Fatty Acids Eicosapentaenoic and Docosahexaenoic Acids in the Treatment of Major Depression and Alzheimer’s Disease: Acting Separately or Synergistically?. Prog. Lipid Res..

[178] Chhetry B.T., Hezghia A., Miller J.M., Lee S., Rubin-Falcone H., Cooper T.B., Oquendo M.A., Mann J.J., Sublette M.E. (2016). Omega-3 Polyunsaturated Fatty Acid Supplementation and White Matter Changes in Major Depression. J. Psychiatr. Res..

[179] Sánchez-Villegas A., Álvarez-Pérez J., Toledo E., Salas-Salvadó J., Ortega-Azorín C., Zomeño M.D., Vioque J., Martínez J.A., Romaguera D., Pérez-López J. (2018). Seafood Consumption, Omega-3 Fatty Acids Intake, and Life-Time Prevalence of Depression in the PREDIMED-Plus Trial. Nutrients.

[180] Twenge J.M., Gentile B., DeWall C.N., Ma D., Lacefield K., Schurtz D.R. (2010). Birth Cohort Increases in Psychopathology among Young Americans, 1938-2007: A Cross-Temporal Meta-Analysis of the MMPI. Clin. Psychol. Rev..

[181] Logan A.C., Jacka F.N. (2014). Nutritional Psychiatry Research: An Emerging Discipline and Its Intersection with Global Urbanization, Environmental Challenges and the Evolutionary Mismatch. J. Physiol. Anthropol..

[182] Hibbeln J.R., Salem N. (1995). Dietary Polyunsaturated Fatty Acids and Depression: When Cholesterol Does Not Satisfy. Am. J. Clin. Nutr..

[183] McNamara R.K., Hahn C.G., Jandacek R., Rider T., Tso P., Stanford K.E., Richtand N.M. (2007). Selective Deficits in the Omega-3 Fatty Acid Docosahexaenoic Acid in the Postmortem Orbitofrontal Cortex of Patients with Major Depressive Disorder. Biol. Psychiatry.

[184] McNamara R.K., Nandagopal J.J., Strakowski S.M., DelBello M.P. (2010). Preventative Strategies for Early-Onset Bipolar Disorder: Towards a Clinical Staging Model. CNS Drugs.

[185] Grosso G., Pajak A., Marventano S., Castellano S., Galvano F., Bucolo C., Drago F., Caraci F. (2014). Role of Omega-3 Fatty Acids in the Treatment of Depressive Disorders: A Comprehensive Meta-Analysis of Randomized Clinical Trials. PLoS ONE.

[186] Sublette M.E., Galfalvy H.C., Hibbeln J.R., Keilp J.G., Malone K.M., Oquendo M.A., Mann J.J. (2014). Polyunsaturated Fatty Acid Associations with Dopaminergic Indices in Major Depressive Disorder. Int. J. Neuropsychopharmacol..

[187] Kelaiditis C.F., Gibson E.L., Dyall S.C. (2023). Effects of Long-Chain Omega-3 Polyunsaturated Fatty Acids on Reducing Anxiety and/or Depression in Adults; A Systematic Review and Meta-Analysis of Randomised Controlled Trials. Prostaglandins Leukot. Essent. Fat. Acids.

[188] Song J., Ma W., Gu X., Zhao L., Jiang J., Xu Y., Zhang L., Zhou M., Yang L. (2019). Metabolomic Signatures and Microbial Community Profiling of Depressive Rat Model Induced by Adrenocorticotrophic Hormone. J. Transl. Med..

[189] Nierenberg A.A., Agustini B., Köhler-Forsberg O., Cusin C., Katz D., Sylvia L.G., Peters A., Berk M. (2023). Diagnosis and Treatment of Bipolar Disorder: A Review. JAMA.

[190] Rutkofsky I.H., Khan A.S., Sahito S., Kumar V. (2017). The Psychoneuroimmunological Role of Omega-3 Polyunsaturated Fatty Acids in Major Depressive Disorder and Bipolar Disorder. Adv. Mind Body Med..

[191] Mcnamara R.K., Jandacek R., Tso P., Blom T.J., Welge J.A., Strawn J.R., Adler C.M., Strakowski S.M., Delbello M.P. (2016). Adolescents with or at Ultra-High Risk for Bipolar Disorder Exhibit Erythrocyte Docosahexaenoic Acid and Eicosapentaenoic Acid Deficits: A Candidate Prodromal Risk Biomarker. Early Interv. Psychiatry.

[192] Bach B., Kramer U., Doering S., di Giacomo E., Hutsebaut J., Kaera A., De Panfilis C., Schmahl C., Swales M., Taubner S. (2022). The ICD-11 Classification of Personality Disorders: A European Perspective on Challenges and Opportunities. Borderline Pers. Disord. Emot. Dysregul..

[193] Gajos J.M., Beaver K.M. (2016). The Effect of Omega-3 Fatty Acids on Aggression: A Meta-Analysis. Neurosci. Biobehav. Rev..

[194] Bègue L., Zaalberg A., Shankland R., Duke A., Jacquet J., Kaliman P., Pennel L., Chanove M., Arvers P., Bushman B.J. (2018). Omega-3 Supplements Reduce Self-Reported Physical Aggression in Healthy Adults. Psychiatry Res..

[195] Choy O., Raine A. (2018). Omega-3 Supplementation as a Dietary Intervention to Reduce Aggressive and Antisocial Behavior. Curr. Psychiatry Rep..

[196] Zanarini M.C., Frankenburg F.R. (2003). Omega-3 Fatty Acid Treatment of Women with Borderline Personality Disorder: A Double-Blind, Placebo-Controlled Pilot Study. Am. J. Psychiatry.

[197] Lieb K., Völlm B., Rücker G., Timmer A., Stoffers J.M. (2010). Pharmacotherapy for Borderline Personality Disorder: Cochrane Systematic Review of Randomised Trials. Br. J. Psychiatry.

[198] Johnson K.V.-A. (2020). Gut Microbiome Composition and Diversity Are Related to Human Personality Traits. Hum. Microb. J..

[199] Vernice N.A., Shah N., Lam E., Herd P., Reiss A.B., Kasselman L.J. (2020). The Gut Microbiome and Psycho-Cognitive Traits. Prog. Mol. Biol. Transl. Sci..

[200] Anderson G. (2020). Pathoetiology and Pathophysiology of Borderline Personality: Role of Prenatal Factors, Gut Microbiome, Mu- and Kappa-Opioid Receptors in Amygdala-PFC Interactions. Prog. Neuropsychopharmacol. Biol. Psychiatry.

[201] Cullen K.R., Vizueta N., Thomas K.M., Han G.J., Lim K.O., Camchong J., Mueller B.A., Bell C.H., Heller M.D., Schulz S.C. (2011). Amygdala Functional Connectivity in Young Women with Borderline Personality Disorder. Brain Connect..

[202] Rössler H., Flasbeck V., Gatermann S., Brüne M. (2022). Alterations of the Gut Microbiota in Borderline Personality Disorder. J. Psychosom. Res..

[203] Ravindran A.V., Balneaves L.G., Faulkner G., Ortiz A., McIntosh D., Morehouse R.L., Ravindran L., Yatham L.N., Kennedy S.H., Lam R.W. (2016). Canadian Network for Mood and Anxiety Treatments (CANMAT) 2016 Clinical Guidelines for the Management of Adults with Major Depressive Disorder: Section 5. Complementary and Alternative Medicine Treatments. Can. J. Psychiatry.

[204] Appleton K.M., Sallis H.M., Perry R., Ness A.R., Churchill R. (2015). Omega-3 Fatty Acids for Depression in Adults. Cochrane Database Syst. Rev..

[205] Appleton K.M., Sallis H.M., Perry R., Ness A.R., Churchill R. (2016). ω-3 Fatty Acids for Major Depressive Disorder in Adults: An Abridged Cochrane Review. BMJ Open.

[206] Hallahan B., Ryan T., Hibbeln J.R., Murray I.T., Glynn S., Ramsden C.E., SanGiovanni J.P., Davis J.M. (2016). Efficacy of Omega-3 Highly Unsaturated Fatty Acids in the Treatment of Depression. Br. J. Psychiatry.

[207] Mocking R.J.T., Harmsen I., Assies J., Koeter M.W.J., Ruhé H.G., Schene A.H. (2016). Meta-Analysis and Meta-Regression of Omega-3 Polyunsaturated Fatty Acid Supplementation for Major Depressive Disorder. Transl. Psychiatry.

[208] Schefft C., Kilarski L.L., Bschor T., Köhler S. (2017). Efficacy of Adding Nutritional Supplements in Unipolar Depression: A Systematic Review and Meta-Analysis. Eur. Neuropsychopharmacol..

[209] Iqbal A.Z., Wu S.-K., Zailani H., Chiu W.-C., Liu W.-C., Su K.-P., Lee S.-D. (2023). Effects of Omega-3 Polyunsaturated Fatty Acids Intake on Vasomotor Symptoms, Sleep Quality and Depression in Postmenopausal Women: A Systematic Review. Nutrients.

[210] Gabriel F.C., Oliveira M., Martella B.D.M., Berk M., Brietzke E., Jacka F.N., Lafer B. (2023). Nutrition and Bipolar Disorder: A Systematic Review. Nutr. Neurosci..

[211] Karaszewska D.M., Ingenhoven T., Mocking R.J.T. (2021). Marine Omega-3 Fatty Acid Supplementation for Borderline Personality Disorder. J. Clin. Psychiatry.

[212] Chang J.P.-C., Tseng P.-T., Zeng B.-S., Chang C.-H., Su H., Chou P.-H., Su K.-P. (2023). Safety of Supplementation of Omega-3 Polyunsaturated Fatty Acids: A Systematic Review and Meta-Analysis of Randomized Controlled Trials. Adv. Nutr..

[213] Ng Q.X., Loke W., Venkatanarayanan N., Lim D.Y., Soh A.Y.S., Yeo W.S. (2019). A Systematic Review of the Role of Prebiotics and Probiotics in Autism Spectrum Disorders. Medicina.

[214] Tan Q., Orsso C.E., Deehan E.C., Kung J.Y., Tun H.M., Wine E., Madsen K.L., Zwaigenbaum L., Haqq A.M. (2021). Probiotics, Prebiotics, Synbiotics, and Fecal Microbiota Transplantation in the Treatment of Behavioral Symptoms of Autism Spectrum Disorder: A Systematic Review. Autism Res..

[215] Song J., Zhou B., Kan J., Liu G., Zhang S., Si L., Zhang X., Yang X., Ma J., Cheng J. (2022). Gut Microbiota: Linking Nutrition and Perinatal Depression. Front. Cell. Infect. Microbiol..

[216] Martínez-González A.E., Andreo-Martínez P. (2020). Prebiotics, Probiotics and Fecal Microbiota Transplantation in Autism: A Systematic Review. Rev. Psiquiatr. Salud Ment..

[217] Clapp M., Aurora N., Herrera L., Bhatia M., Wilen E., Wakefield S. (2017). Gut Microbiota’s Effect on Mental Health: The Gut-Brain Axis. Clin. Pract..

[218] McKean J., Naug H., Nikbakht E., Amiet B., Colson N. (2017). Probiotics and Subclinical Psychological Symptoms in Healthy Participants: A Systematic Review and Meta-Analysis. J. Altern. Complement. Med..

[219] Smith K.S., Greene M.W., Babu J.R., Frugé A.D. (2021). Psychobiotics as Treatment for Anxiety, Depression, and Related Symptoms: A Systematic Review. Nutr. Neurosci..

[220] Liu R.T., Walsh R.F.L., Sheehan A.E. (2019). Prebiotics and Probiotics for Depression and Anxiety: A Systematic Review and Meta-Analysis of Controlled Clinical Trials. Neurosci. Biobehav. Rev..

[221] Zagórska A., Marcinkowska M., Jamrozik M., Wiśniowska B., Paśko P. (2020). From Probiotics to Psychobiotics—The Gut-Brain Axis in Psychiatric Disorders. Benef. Microbes.

[222] Sanada K., Nakajima S., Kurokawa S., Barceló-Soler A., Ikuse D., Hirata A., Yoshizawa A., Tomizawa Y., Salas-Valero M., Noda Y. (2020). Gut Microbiota and Major Depressive Disorder: A Systematic Review and Meta-Analysis. J. Affect. Disord..

[223] Grau-Del Valle C., Fernández J., Solá E., Montoya-Castilla I., Morillas C., Bañuls C. (2023). Association between Gut Microbiota and Psychiatric Disorders: A Systematic Review. Front. Psychol..

[224] Fond G.B., Lagier J.-C., Honore S., Lancon C., Korchia T., Sunhary De Verville P.-L., Llorca P.-M., Auquier P., Guedj E., Boyer L. (2020). Microbiota-Orientated Treatments for Major Depression and Schizophrenia. Nutrients.

[225] Knuesel T., Mohajeri M.H. (2021). The Role of the Gut Microbiota in the Development and Progression of Major Depressive and Bipolar Disorder. Nutrients.

[226] Alli S.R., Gorbovskaya I., Liu J.C.W., Kolla N.J., Brown L., Müller D.J. (2022). The Gut Microbiome in Depression and Potential Benefit of Prebiotics, Probiotics and Synbiotics: A Systematic Review of Clinical Trials and Observational Studies. Int. J. Mol. Sci..

[227] Halemani K., Shetty A.P., Thimmappa L., Issac A., Dhiraaj S., Radha K., Mishra P., Mathias E.G. (2023). Impact of Probiotic on Anxiety and Depression Symptoms in Pregnant and Lactating Women and Microbiota of Infants: A Systematic Review and Meta-Analysis. J. Glob. Health.

[228] Ng Q.X., Peters C., Ho C.Y.X., Lim D.Y., Yeo W.-S. (2018). A Meta-Analysis of the Use of Probiotics to Alleviate Depressive Symptoms. J. Affect. Disord..

[229] Le Morvan de Sequeira C., Hengstberger C., Enck P., Mack I. (2022). Effect of Probiotics on Psychiatric Symptoms and Central Nervous System Functions in Human Health and Disease: A Systematic Review and Meta-Analysis. Nutrients.

[230] Ng Q.X., Lim Y.L., Yaow C.Y.L., Ng W.K., Thumboo J., Liew T.M. (2023). Effect of Probiotic Supplementation on Gut Microbiota in Patients with Major Depressive Disorders: A Systematic Review. Nutrients.

[231] Ciobanu A.M., Petrescu C., Anghele C., Manea M.C., Ciobanu C.A., Petrescu D.M., Antonia M.O., Riga S. (2023). Severe Vitamin D Deficiency-A Possible Cause of Resistance to Treatment in Psychiatric Pathology. Medicina.

[232] Seiler N., Tsiglopoulos J., Keem M., Das S., Waterdrinker A. (2022). Prevalence of Vitamin D Deficiency among Psychiatric Inpatients: A Systematic Review. Int. J. Psychiatry Clin. Pract..

[233] Cui X., McGrath J.J., Burne T.H.J., Eyles D.W. (2021). Vitamin D and Schizophrenia: 20 Years On. Mol. Psychiatry.

[234] Wang Z., Ding R., Wang J. (2020). The Association between Vitamin D Status and Autism Spectrum Disorder (ASD): A Systematic Review and Meta-Analysis. Nutrients.

[235] Tirani S.A., Balali A., Askari G., Saneei P. (2023). Maternal Serum 25-Hydroxy Vitamin D Levels and Risk of Autism Spectrum and Attention-Deficit Hyperactivity Disorders in Offspring: A Systematic Review and Dose-Response Meta-Analysis. Psychiatry Res..

[236] Mikola T., Marx W., Lane M.M., Hockey M., Loughman A., Rajapolvi S., Rocks T., O’Neil A., Mischoulon D., Valkonen-Korhonen M. (2023). The Effect of Vitamin D Supplementation on Depressive Symptoms in Adults: A Systematic Review and Meta-Analysis of Randomized Controlled Trials. Crit. Rev. Food Sci. Nutr..

[237] Srifuengfung M., Srifuengfung S., Pummangura C., Pattanaseri K., Oon-Arom A., Srisurapanont M. (2023). Efficacy and Acceptability of Vitamin D Supplements for Depressed Patients: A Systematic Review and Meta-Analysis of Randomized Controlled Trials. Nutrition.

[238] Sarris J., Ravindran A., Yatham L.N., Marx W., Rucklidge J.J., McIntyre R.S., Akhondzadeh S., Benedetti F., Caneo C., Cramer H. (2022). Clinician Guidelines for the Treatment of Psychiatric Disorders with Nutraceuticals and Phytoceuticals: The World Federation of Societies of Biological Psychiatry (WFSBP) and Canadian Network for Mood and Anxiety Treatments (CANMAT) Taskforce. World J. Biol. Psychiatry.

